# Novel Genes and Pathways Modulated by Syndecan-1: Implications for the Proliferation and Cell-Cycle Regulation of Malignant Mesothelioma Cells

**DOI:** 10.1371/journal.pone.0048091

**Published:** 2012-10-29

**Authors:** Tünde Szatmári, Filip Mundt, Ghazal Heidari-Hamedani, Fang Zong, Elena Ferolla, Andrey Alexeyenko, Anders Hjerpe, Katalin Dobra

**Affiliations:** 1 Department of Laboratory Medicine, Division of Pathology, Karolinska Institutet, Stockholm, Sweden; 2 Science for Life Laboratory, School of Biotechnology, KTH Royal Institute of Technology, Stockholm, Sweden; Universität Heidelberg, Germany

## Abstract

Malignant pleural mesothelioma is a highly malignant tumor, originating from mesothelial cells of the serous cavities. In mesothelioma the expression of syndecan-1 correlates to epithelioid morphology and inhibition of growth and migration. Our previous data suggest a complex role of syndecan-1 in mesothelioma cell proliferation although the exact underlying molecular mechanisms are not completely elucidated. The aim of this study is therefore to disclose critical genes and pathways affected by syndecan-1 in mesothelioma; in order to better understand its importance for tumor cell growth and proliferation. We modulated the expression of syndecan-1 in a human mesothelioma cell line via both overexpression and silencing, and followed the transcriptomic responses with microarray analysis. To project the transcriptome analysis on the full-dimensional picture of cellular regulation, we applied pathway analysis using Ingenuity Pathway Analysis (IPA) and a novel method of network enrichment analysis (NEA) which elucidated signaling relations between differentially expressed genes and pathways acting via various molecular mechanisms. Syndecan-1 overexpression had profound effects on genes involved in regulation of cell growth, cell cycle progression, adhesion, migration and extracellular matrix organization. In particular, expression of several growth factors, interleukins, and enzymes of importance for heparan sulfate sulfation pattern, extracellular matrix proteins and proteoglycans were significantly altered. Syndecan-1 silencing had less powerful effect on the transcriptome compared to overexpression, which can be explained by the already low initial syndecan-1 level of these cells. Nevertheless, 14 genes showed response to both up- and downregulation of syndecan-1. The “cytokine – cytokine-receptor interaction”, the TGF-β, EGF, VEGF and ERK/MAPK pathways were enriched in both experimental settings. Most strikingly, nearly all analyzed pathways related to cell cycle were enriched after syndecan-1 silencing and depleted after syndecan-1 overexpression. Syndecan-1 regulates proliferation in a highly complex way, although the exact contribution of the altered pathways necessitates further functional studies.

## Introduction

Syndecans are a family of cell surface heparan sulfate proteoglycans (HSPGs) with an extracellular domain carrying glycosaminoglycan (GAG) side chains, a transmembrane domain and a short cytoplasmic domain [1]. The syndecan family contains four members (syndecan 1–4); and there is a distinct pattern of syndecan expression and GAG modification that characterizes individual cell types and tissues. A number of studies have shown that syndecans play critical roles in cellular processes including differentiation, cell adhesion [2], [3], cytoskeletal organization, cell spreading and migration [4], [5], [6], infiltration, angiogenesis [7], [8] and proliferation of various malignant tumors [8], [9], [10]. Syndecans exert these functions partly through their GAG chains, mainly heparan sulfate, but recent studies show that different domains of the core protein have distinct roles as well [3], [11]. Syndecan-1 is overexpressed in some tumor types, whereas suppressed in others [12]. It is well known that the expression of syndecans is strictly regulated in a tissue dependent manner in many epithelial tumors, where syndecan-1 is the main syndecan. In mesenchymal tumors its expression level is generally low, hence only few studies have addressed syndecan-1’s role and regulation in these tumors [13], [14].

The mesothelium is a mesenchymal tissue with an inherited ability to differentiate across the epithelial-mesenchymal axis. This ability to transdifferentiate is also preserved in malignant mesothelioma which can arise in this tissue as a consequence of asbestos exposure [15]. The differentiation of these aggressive tumors involves syndecans [16], and particularly syndecan**-**1 [17], which modulates a number of growth-factor – growth-factor receptor interactions, thus acting as a signaling co-receptor [10]. We have previously shown that overexpression of syndecan-1 in malignant mesothelioma correlates with epithelioid differentiation and inhibition of tumor growth [13] and migration [14]. Furthermore, the presence of syndecan-1 implies a better prognosis of malignant mesothelioma [17]. Our previous studies also suggest that the ratio between syndecan-1 and syndecan-2 may distinguish a primary malignant mesothelioma from a metastatic adenocarcinoma [16], [18]. This implies complex regulatory mechanisms, which are tissue and/or tumor type-specific, and at least partly dependent upon the tumor’s interplay with the surrounding matrix.

The objective of this study is to reveal genes and pathways influenced by syndecan-1 in malignant pleural mesothelioma for a better understanding of its importance for the malignant behavior of this mesenchymal tumor. For this purpose we modulated syndecan-1 expression in a human malignant mesothelioma cell line and performed microarray analysis to investigate the effects of syndecan-1 overexpression and silencing on general transcriptional level. Our previous data show that overexpression of syndecan-1 inhibits proliferation of malignant mesothelioma; in this paper we also investigated the effect of syndecan-1 silencing on the proliferation rate and cell cycle distribution of these cells. In particular, we aim to characterize the molecular events underlying the growth modulatory effect of syndecan-1 and to identify critical factors and pathways dependent on syndecan-1, focusing on cell-cycle regulation and features related to proliferation. While analyzing the global transcriptome response, it is crucial to see both comprehensive changes and pivotal functional mechanisms behind them. To this end, we described the transcription profiles of individual genes in three different ways, using: 1) the conventional Gene Set Enrichment Analysis (GSEA [19]) based on Gene Ontology(GO) categories, and two network-based methods: 2) Ingenuity Pathway Analyzer (IPA, Ingenuity® Systems, www.ingenuity.com), which performs GSEA on network modules of differentially expressed (DE) genes and 3) a novel method of network enrichment analysis (NEA, [20]) that finds pathway relations of DE genes irrespective of the network modularity and does not depend on their pathway annotations.

Generally for functional analyses of novel gene sets (altered gene sets, AGS), they are matched to different gene groups with previously known functional attributes (functional gene sets, FGS). In the conventional GSEA, the information is summarized by finding over-representation of certain FGSs in the list of AGS genes. This approach is simple and convincing, although entirely ignores functional relations between AGS genes themselves and between AGS and outside pathways. Hence, it is desirable to go beyond a simple overlap between AGS and members of FGS. For this purpose, IPA attempts to identify differentially expressed genes grouped in compact modules in the network. However, DE genes not necessarily group like this. In contrast to other network methods, the NEA does not expect any ready modules in the network and considers functional links between *any* genes of AGS and FGS in the whole gene interaction network. In other words, it uses available network links scattered over the network to test enrichment hypotheses of functional associations between an experimentally defined gene set and known pathways and biological processes. Hence, NEA acts in the most straightforward and robust GSEA-like manner with the difference that, unlike conventional GSEA, it employs DE genes which are not necessarily members of any already known functional category; but they are connected to such members in the network. Due to the availability of gene network links to nearly every gene, the sensitivity of the method exceeds that of GSEA around 5–10 fold [20].

In the following, we systematically apply the GSEA, IPA, and NEA to our research problem. By combining these methods we highlight the most crucial biological processes controlled by syndecan-1 in malignant mesothelioma.

## Materials and Methods

### Cell Culture Conditions

Human malignant mesothelioma cells (STAV-AB) were grown in RPMI 1640 medium supplemented with 10% human AB serum, 1% L-Glutamine and 25 mM HEPES buffer under standardized incubation conditions, in humidified atmosphere containing 5% (v/v) CO_2_ at 37°C. The cells display epithelioid morphology and express low endogenous syndecan-1 [16].

### Syndecan-1 Overexpression

Syndecan-1 was stably overexpressed by transfection with a pEGFP-N1 plasmid carrying the human full-length syndecan-1 gene. Transfection was performed using Effectene Transfection Reagent (Qiagen GmbH, Hilden, Germany). The plasmid and subsequent stable transfection of STAV-AB cells have previously been described in detail by us in a recent publication [13]. Cells transfected with the pEGFP-N1 vector were used as negative control.

### Syndecan-1 Silencing

Three hundred thousand STAV-AB cells/well were seeded and after one day transfected using Lipofectamine™ 2000 (Invitrogen, Carlsbad, CA, USA). Three siRNA constructs specific for syndecan-1 were used (Ambion®, Inc. Stockholm, Sweden) (Table S1) with an optimized concentration of 40 nM. Scrambled siRNA, with no target mRNA, was used as negative control. Syndecan-1 specific siRNA or scrambled control siRNA and lipofectamine were diluted in antibiotics- and serum- free medium according to the manufacturer’s instructions and incubated for 25 minutes. The different mediums containing the siRNA-lipofectamine complexes were then added separately to the cells and incubated at 37°C and 5% CO_2_. After 24 or 48 hours the samples were harvested and RNA was extracted. Experiments were performed in triplicates or more.

### RNA Isolation

Total RNA was isolated from sub-confluent cells, using the High Pure RNA Isolation Kit (Roche, Mannheim, Germany) according to the manufacturer’s protocol. The yield and purity of the RNA were determined spectrophotometrically by measuring the UV absorbances at 260 and 280 nm with a NanoDrop spectrophotometer (NanoDrop Technologies Inc.).

### Validation

#### a. Quantitative real time polymerase chain reaction (RT-PCR)

Verification of the syndecan-1 overexpression and silencing, as well as validation of the Affimetryx results, were done by quantitative RT-PCR. cDNA synthesis was performed by reverse transcription of 2 µg RNA using First Strand cDNA Synthesis Kit (Amersham Pharmacia Biotech., Little Chalfont, Buckinghamshire, England). RT-PCR was performed with the Platinum® SybrGreen qPCR SuperMix-UDG kit (Invitrogen®) using DNA-polymerase with a set of sense/antisense primers (CyberGene AB, Sweden) for all target genes (see Table S2). The primers were designed by us, using gene sequences from GeneBank (NCBI) with exception of syndecan-1 [21] and GAPDH [22]. All reactions were performed in triplicate, using a total volume of 10 µL/well, with primer concentration of 200 nM, in an iCycler machine (CFX96™ Real Time PCR Detection System, BioRAD Hercules, CA, USA). Analysis was done with Bio-Rad CFX Manager Software 2.0 (BioRad Laboratories 2008). The quantity of each target was normalized to GAPDH as reference gene and to the corresponding controls (vector for syndecan-1 overexpression and scrambled siRNA for syndecan-1 silencing), respectively. Normalization was done by delta delta Ct method, by first determining ΔCt as average Ct_target_gene_-average Ct_GAPDH_, then ΔΔCt as ΔCt_sample_-ΔCt_control_. Relative expression was calculated as 2^−ΔΔCt^; fold-change was represented by relative expression if >1 and -1/relative expression if <1. Data were presented as mean values of at least three independent experiments.

#### b. Flow cytometry

Flow cytometry was performed for confirmation of syndecan-1 modulation on protein level. Cells modulated for syndecan-1 or their respective controls (vector for overexpression and scrambled control for silencing) were detached using 5 mM EDTA in phosphate-buffered saline (PBS) and were fixed in 1% formaldehyde. Cells were permeabilised with 0.1% saponin and 1% BSA in PBS for 10 min. Cells were stained with PE-conjugated specific antibody against syndecan-1 (CD138, clone B-A38, Ref. no. IQP-153R, IQ® PRODUCTS, Groningen, The Netherlands) for 15 minutes in dark. The corresponding isotype IgG1 control (Ref. no. IQP-191R, IQ® PRODUCTS) was used as a negative control. FACS analysis was performed using FACS Calibur Cytometer (Becton Dickinson, San Jose, CA, USA). Results were analyzed with Cell Quest Pro software. Three independent experiments were performed, analyzing at least 10,000 cells for each sample.

#### c. Proteome profiler arrays

In order to validate changes caused by syndecan-1 overexpression at protein level, Proteome Profiler Antibody Array (R&D Systems, Inc.) was used. Supernatants from cells overexpressing syndecan-1 and corresponding vector control were collected by centrifugation (400 G, 5 minutes) and the volume was normalized to the cell number. The relative expression of proteins of interest was determined in each sample according to the manufacturer’s instruction. Briefly, nitrocellulose membranes spotted with primary antibodies against 55 proteins were blocked and supernatants, mixed with a cocktail of biotinylated detection antibodies, were added to the membranes and incubated overnight at 4°C. Streptavidin-horseradish peroxidase (HRP) was then added to the membranes and incubated for 30 minutes before chemiluminescence detection reagents were added in equal volumes for approximately. 1 minute. Dot blots were registered with CCD camera (FluorChem™ SP, Alpha Innotech, USA). The average pixel density of duplicate spots on the membrane was determined using ImageJ software (Rasband, W.S., ImageJ, U. S. National Institutes of Health, Bethesda, Maryland, USA, http://rsb.info.nih.gov/ij/, 1997–2008). After background subtraction the relative amounts of individual proteins were calculated.

### Functional Assays

#### a. Cell proliferation

Syndecan-1 was silenced using 3*10^5^ cells, as described above. Second day silenced cells or scrambled controls were harvested and equal volumes were reseeded in 96 well plates. Cell proliferation was assessed by Cell Proliferation Reagent WST-1 (Roche Diagnostics Scandinavia AB, Bromma, Sweden) at 0, 24, 48 and 72 hours after silencing, according to the manufacturer’s instruction. Briefly, cells were incubated with 1/10 (v/v) WST1 reagent for 3 hours at 37°C. Samples were analyzed using a Spectramax spectrophotometer at 450 nm with background subtraction at 630 nm. Cell numbers were obtained by interpolating absorbance values with a standard curve. The 72 hours absorbance values were not used for the analysis as they were saturated. Three independent experiments were performed, each containing quadruplicates. To determine statistical significance a two**-way ANOVA with Bonferroni’s posttest was performed, using GraphPad Prism software.** Doubling time was calculated from the logarithmic phase of the growth curve (Roth V. 2006 http://www.doubling-time.com/compute.php).

#### b. Analysis of cell cycle distributions

Twenty-four and forty-eight hours after syndecan-1 silencing, cells silenced for syndecan-1 or cells transfected with scrambled control siRNA were harvested using 5 mM EDTA and fixed overnight in cold ethanol. Cells were stained using propidium iodide (PI)/RNase solution (PI concentration at 50 µg/mL and RNase concentration at 100 µg/mL) and incubated for 30 min in 37°C in dark. Cell cycle analysis was performed by measuring the amount of incorporated PI reflecting the DNA content of the cells, using FACS Calibur Cytometer and CELLQuest Pro software. Three independent experiments were performed, gating 20,000 cells in each sample.

#### c. Apoptosis assay

Cells silenced for syndecan-1 and scrambled controls were stained at 24 and 48 hours after syndecan-1 silencing with Annexin-V-FITC and Propidium iodide (PI), using Annexin-V-FITC apoptosis detection kit (BD Biosciences, San Diego, CA) according to the manufacturer’s instructions. Briefly, cells were washed with cold PBS and resuspended in binding buffer in concentration of 10^6^ cells/mL. 5 µl of both Annexin V-FITC and PI were added to 10^5^ cells and incubated for 15 min at room temperature in dark. 400 µl of binding buffer was added to cells and analyzed by FACS Calibur Cytometer. Data from 10,000 events in each sample were collected and data was analyzed using CELLQuest Pro software. Three independent experiments were performed.

### Statistical Analysis

Statistical analysis was performed using GraphPad Prism version 5.02 for Windows, (GraphPad Software, San Diego California USA, www.graphpad.com). Unless otherwise stated, the difference between the mean values of cells modified for syndecan-1 and control cells were analyzed using two tailed student’s t-test. Statistical significance (*) was considered at p<0.05. Standard deviation (SD) is represented as errors bars on figures or as numerical values in text or tables.

### Microarray Analysis

We analyzed the individual transcriptome of STAV-AB mesothelioma cells with overexpressed and silenced syndecan-1 compared to their corresponding controls. Microarray analysis was performed using the GeneChip® Human Gene 1.0 ST Array (Affymetrix Inc., Santa Clara, CA, USA) that offers whole-transcript coverage. Each of the 28,869 genes was represented on the array with around 26 probes spread along the full length of the gene, providing a complete and accurate coverage of gene expression. Background was estimated using a set of approximately 20,000 generic background probes. Standard poly-A controls and hybridization controls were also represented on the array to allow troubleshooting along the entire experimental process. Target synthesis and hybridization was performed in the Affymetrix core facility (Novum, Karolinska Institutet, Stockholm, Sweden). The raw data has been deposited in the MIAME compliant database Gene Expression Omnibus (accession numbers GSE21401 and GSE37843). The image analysis and data pre-processing was performed by Affymetrix Gene Chip Command Console: background correction was done with PM-GCBG method (subtracting a GC-content specific background), data were normalized with Global Median method and raw intensity values were summarized with PLIER (Probe Logarithmic Intensity Error). After the diffrential expression analysis probeset IDs were converted to HUPO gene symbols, which we used throughout the analysis to denote the genes.

### Differential Gene Expression

Differential gene expression (DE) values were determined by OCplus package within R software [23]. To account for biological variability between the three cell isolates, OCplus analyses were done on triplicates with paired t-test. Within the same package, multiple testing correction converted p-values (the probability of a non-DE gene to appear as DE in the statistical test) to false discovery rates (q-values, the probability of the statistical finding for the given gene to be genuinely false) (Figure S1). Differentially expressed genes were ranked by fold-change relating a syndecan-1 modulated sample to its corresponding control. A fold change (FC, i. e. the ratio of expression values between a syndecan-1 modulation and respective control) cut-off of <−1.5 or >1.5– and a q-value of <0.05 was set to define a transcript as significantly up- or downregulated.

### Molecular Pathway Analysis

Molecular pathway analysis was performed to reveal possible involvement of genes with specific biological functions following both syndecan-1 overexpression and silencing. This allows the investigation of functional relations between differentially expressed genes, especially when summarizing small changes in many related genes.

#### a. Ingenuity pathway analysis (IPA)

A dataset containing gene identifiers and corresponding expression values were uploaded to the Ingenuity Pathway Analysis software (IPA, Ingenuity® Systems, www.ingenuity.com). The above defined cut-off of fold-change and q-values were used to identify transcripts as significantly up/downregulated. The transcripts generated by this approach (Network Eligible Transcripts) were overlaid in the Ingenuity Knowledge Database and networks were algorithmically generated, based on their connectivity. The Ingenuity Knowledge Database was created from manually curated literature searches and peer-reviewed for accuracy by subject matter experts. Specific data on the number of molecules and interactions is not reported [24]. Molecules from the dataset that met the above mentioned cutoff criteria and were associated with biological functions and/or diseases in the Ingenuity Knowledge Database were considered for the analysis. The Functional Analysis identified significantly affected functions and pathways. To further investigate specific networks with a role in cell proliferation and cell cycle regulation, the groups of genes identified by IPA as related to these functional groups (“cell growth and proliferation” and “cell cycle”) were uploaded separately into the Ingenuity Network Explorer and network linkages were identified based on published literature in the Ingenuity Knowledge Database.

#### b. Network enrichment analysis (NEA)

Network enrichment defines statistically over-represented functional gene sets (most often pathways or gene ontology categories) in the list of an altered gene set from a microarray experiment. The idea of network enrichment analysis (NEA) is similar to that of gene set enrichment analysis (GSEA) [25]. The difference is that in NEA relations of differentially expressed genes to functional groups are established in the gene network. Practically, with NEA we quantified the over/under representation of the functional group members among the neighbors in the gene network rather than in the altered gene set (AGS) itself. Hence, the differentially expressed genes (members of AGS) are taken in consideration regardless if they belong or not to already known functional categories.

*Altered gene sets* (AGS) were constructed from lists of differentially expressed genes considering each comparison [“full length syndecan-1” vs. “empty vector” (FL2E) and “silenced syndecan-1” vs. “scrambled control” (SI2NS)] were generated (based on their q-values). Each comparison resulted in two alternative lists: top 100 and top 900 most significantly altered genes.

*Functional gene sets* (FGS) were constructed to characterize altered gene sets by their involvement in known biological processes. For this, we collected lists of genes, members of known pathways and other gene groups of importance in the context of cancer. We used 1,641 functional gene sets derived from the following sources: (i) 9 categories that are closest to the “hallmarks of cancer” [26], [27] were defined as either closest GO terms or gene sets related to known functions of syndecan-1; (ii) all KEGG pathways [28] present in human (as of 21^th^ of April 2010), including 9 cancer pathways; and (iii) gene sets provided as curated gene sets “C2” for gene set enrichment analysis at http://www.broadinstitute.org/gsea/msigdb/genesets.jsp.

To achieve maximal coverage and sensitivity of the analysis, we obtained a *network* for the enrichment analysis by merging the FunCoup network of functional coupling [29] and known links from the curated databases (KEGG pathways [28] and CORUM database of protein complexes [30]), which resulted in a union network of 1,484,166 functional links between 16,302 distinct HUPO gene symbols.

*Network enrichment* was estimated by using NEA Z-scores. The standard Z-score for the biological network connectivity between genes of a novel list *A* and genes of a known functional group *F* was computed from the observed and expected link counts and their standard deviation: 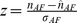
_._

Obtaining values for expected (mean) number 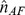 and standard deviation 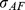 would be affected by different node degree compositions in particular gene sets. To make the analysis unbiased, a network randomization procedure systematically re-wired network nodes, i.e. randomly swapped edges between two nodes at a time, preserving node degrees and the total number of edges in the network. Hence, the expected mean 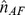 (counted in the same way as the value of *n_AF_*) and standard deviation 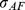
were learned after a sufficient number (50) of random network permutations. Under true null, i.e. in absence of any functional linkages between gene groups, the z-scores were distributed almost normally. Hence, *Z* could be converted to specific NEA p-values (the probability of an irrelevant FGS to appear as related to AGS in the network enrichment analysis) and to false discovery rates (FDR, the probability of the FGS detected as related to AGS to be genuinely irrelevant, i.e. false finding) by standard procedures. The null hypothesis could, however, be rejected when e.g. either one of the two gene sets was small (<5 genes). We thus used the raw Z for ranking results and roughly establishing significance (FDR) thresholds via gene permutation sets, i.e. by recording how frequently randomly compiled gene sets of matched size and individual gene connectivity reached the z-score level.

## Results

### Effect of Syndecan-1 Silencing on Cell Proliferation and Cell Cycle Distribution

**Figure 1:**
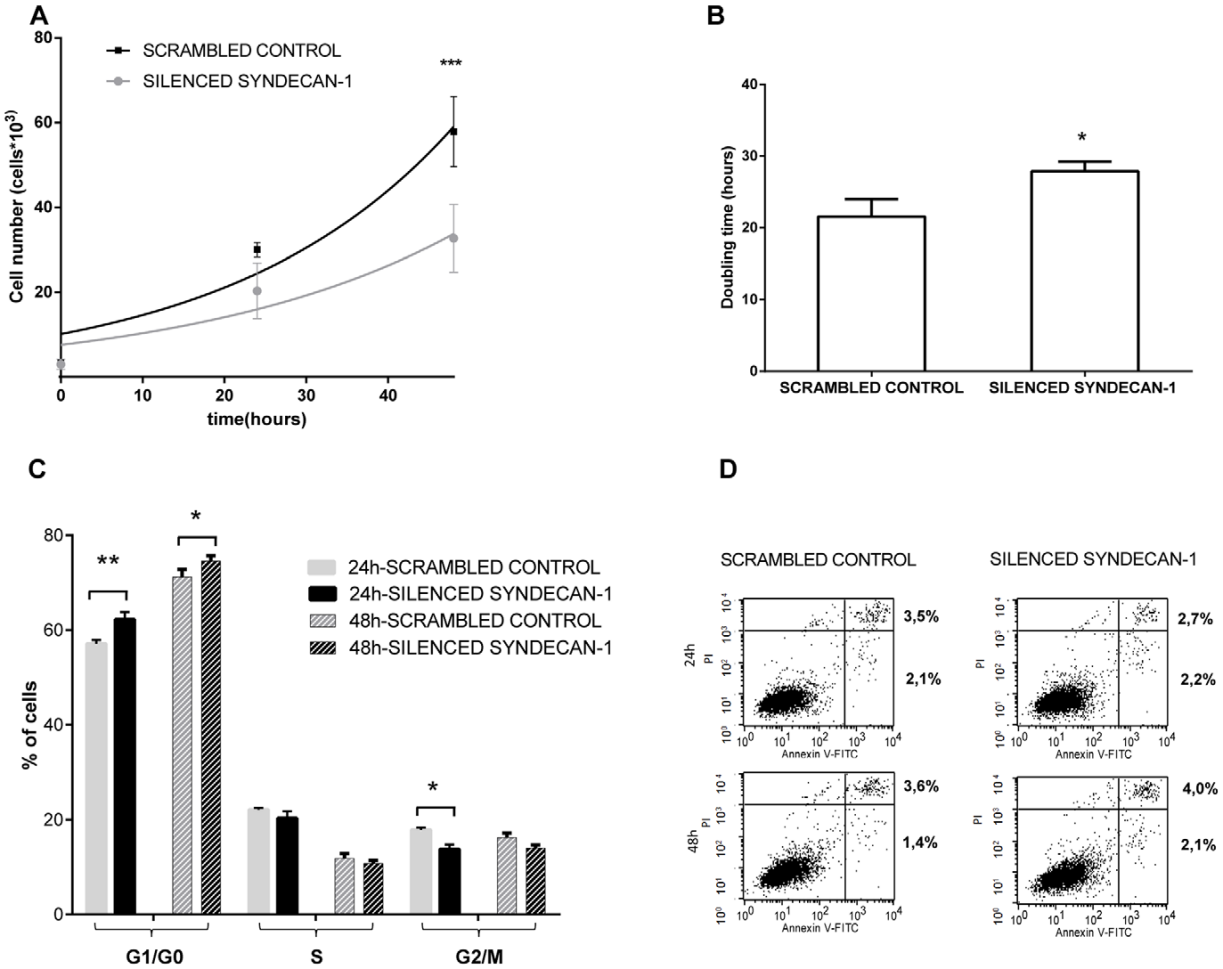
Effect of syndecan-1 silencing on cell growth (A) doubling time (B), cell-cycle distribution (C) and apoptosis (D). (**A**) Proliferation of cells silenced for syndecan-1 and corresponding scrambled control was measured using WST1 proliferation assay. Values are mean of cell number ± SD (n = 3), obtained from three independent experiments with 4 replicates in each. (**B**) Doubling time was calculated from the logarithmic phase of the growth curve. Silencing of syndecan-1 significantly decreased proliferation of mesothelioma cells (*p<0.05, ***p<0.001). (**C**) Cell cycle analysis was performed by propidium iodide staining followed by flow cytometry, 24 and 48 hours after silencing. Columns represents mean percentage of cells in each phase of the cell cycle ± SD (n = 3) (**D**) Apoptosis was measured by flow cytometry using Annexin-V-FITC and Propidium iodide (PI) staining. Cells silenced for syndecan-1 and scrambled controls were stained with PI and Annexin-V, 24 and 48 hours after syndecan-1 silencing. Three independent experiments were performed. Representative Annexin V/PI plots are shown where X axis shows the log of fluorescence intensity of Annexin-V and Y axis demonstrate the log of fluorescence intensity of PI. Cells in the lower left quadrant represent living cells, in the lower right quadrant early apoptotic cells; the upper right and left quadrant show late apoptotic and necrotic cells, respectively. No significant change in apoptosis was recorded. Asterisks indicate statistically significant differences compared to the scrambled control (* p≤0.05, ** p≤0.01, *** p≤0.001). A two-tailed t test was performed to test the statistical significance between cells silenced for syndecan-1 and scrambled control. SD = standard deviation.

Cell proliferation significantly decreased in cells with silenced syndecan-1. This effect was seen 24 hours after transfection (25% of the control) and it was further accentuated at 48 hours (40% of the control) (Figure 1A). Doubling time increased correspondingly from 21.6 hours to 27.9 hours in the syndecan-1 silenced cells compared to the scrambled control (Figure 1B). The cell cycle analysis showed that due to syndecan-1 silencing the amount of G2/M cells was significantly reduced after 24 hours, while an increased number of cells were seen in G0/G1 phase in cells silenced for syndecan-1, compared to scrambled control (Figure 1C). No significant difference was detected in the rate of apoptotic cells in the syndecan-1 silenced cells compared to the scrambled siRNA control (Figure 1D, Table S3).

### Gene Expression Profiling

With a cut-off value of 2 fold change (FC) expression of 1,124 genes was significantly altered in case of syndecan-1 overexpression, whereas only 21 genes were differentially expressed due to syndecan-1 silencing. Thus overexpression of syndecan-1 had a larger effect compared to silencing. With a lower FC cut-off (1.5), we found 2,389 differentially expressed genes in syndecan-1 overexpressed and 103 in silenced cells, respectively. Fourteen genes were concordantly altered by both syndecan-1 overexpression and silencing. ETS-1, TNSF-18, CLIP-4 and FBLN5 were altered in the same direction with syndecan-1 modulation. Interestingly, several proteins were regulated in the same direction regardless of syndecan-1 modulation (see Table 1).

**Table 1:** Genes affected by both syndecan-1 overexpression and silencing.

Gene	Gene name	FC_overexp_	FC_silenced_
**LRRC7**	leucine rich repeat containing 7	**−32.3**	**1.6**
**WDR54**	WD repeat domain 54	**−1.9**	**1.5**
**ETS1**	v-ets erythroblastosis virus E26 oncogene homolog 1 (avian)	**3.9**	**−1.5**
**TNFSF18**	tumor necrosis factor (ligand) superfam. member 18	**3.9**	**−1.6**
**CLIP4**	CAP-GLY-domain-containing linker protein family, member 4	**2.3**	**−1.5**
**FBLN5**	fibulin 5	**1.5**	**−3.4**
**TLN1**	talin 1	**1.8**	**1.9**
**GJA5**	gap junction protein, alpha 5, 40 kDa	**2.0**	**1.8**
**TNXB**	tenascin XB	**7.1**	**1.6**
**ALCAM**	activated leukocyte cell adhesion molecule	**1.7**	**1.6**
**NTSR1**	neurotensin receptor 1 (high affinity)	**1.7**	**1.5**
**NUP62CL**	nucleoporin 62 kDa C-terminal like	**−1.7**	**−1.5**
**EPYC**	epiphycan	**−9.0**	**−1.6**
**SMARCD3**	SWI/SN-rel, matrix-assoc, actin dependent regulator of chromatin, subfamily d, member 3	**−4.1**	**−1.6**

FC_overexp_ represents fold changes for expression levels following syndecan-1 upregulation compared to vector transfected control cells. FC_silenced_ represents fold changes after silencing of syndecan-1 compared to control cells treated with negative/scrambled siRNA. All gene changes are significant at q≤0.05. Fold changes and q-values were calculated using OCplus package in R.

### Most Differentially Expressed Genes

In the syndecan-1 overexpressing cells, the most downregulated genes were functionally heterogeneous. Sulfotransferases and sulfatase1 (SULF1) are genes with role in glycosaminoglycan synthesis and modification, while inhibin A (INHBA) and transforming growth factor-beta 2 (TGFβ2) are cytokines of the TGFβ family. There were also genes involved in adhesion such as densin (LRRC7), desmoplakin (DSP), mucin (MUC16), fibronectin (FN) and nephronectin (NPNT). Among the most upregulated genes we found interleukins and their receptors (IL33, IL8, IL6, IL6R, IL1R1 etc), the intracellular proteoglycan serglycin (SRGN) and alpha-2-macroglobulin (A2M), all with important roles in inflammatory reactions. This group also included the metastasis suppressing protein (MTSS) and neuropilin-2 (NRP2). Neuropilin is a protein earlier associated with cancer progression [31] and shown to be upregulated in mesotheliomas [32] (Table 2). In the cells silenced for syndecan-1, the extent of gene expression modification was more modest. The largest changes included fibulin5 (FBLN5), an extracellular matrix protein antagonistic to fibronectin and affecting proliferation. Other substantially deregulated genes were the leucine-rich-repeat interacting protein (LRRFIP1), which regulates a number of growth factors; the MAPK family DUSP19 and several RNA binding motif proteins (Table 2). There were many transcripts represented without a protein annotation or gene symbol, such as genes encoding for hypothetical proteins whose functions are not yet known.

**Table 2:** Top-ranked list of most down- and upregulated genes following syndecan-1 overexpression and silencing.

Gene	Gene name		FC	
***Syndecan-1 overexpression***				
**IL33**		interleukin 33		**277.0**
**A2M**		alpha-2-macroglobulin		199.2
**IL6**		interleukin 6 (interferon, beta 2)		76.3
**SRGN**		serglycin		52.9
**MTSS1**		metastasis suppressor 1		18.3
**NRP2**		neuropilin 2		18.0
**PDGFRA**		platelet-derived growth factor receptor, alpha		11.13
**IL8**		interleukin 8		10.0
**IL1R1**		interleukin 1 receptor, type I		9.8
**RARRES**		retinoic acid receptor responder 1		9.2
**FN1**		fibronectin 1		−9.1
**GPC6**		glypican 6		−9.3
**MUC16**		mucin 16, cell surface associated		−9.4
**TRIM29**		tripartite motif-containing 29		−9.7
**TGFB2**		transforming growth factor, beta 2		−13.1
**DSP**		desmoplakin		−13,4
**LUM**		lumican		−16,5
**TUBA1A**		tubulin, alpha 1a		−24.6
**ADAMTS5**		ADAM metallopeptidase motif, 5 (aggrecanase-2)		−26.8
**LRRC7**		leucine rich repeat containing 7		−32.3
**SLAMF7**		SLAM family member 7		−49.0
**SULF1**		sulfatase 1		−52.3
**INHBA**		inhibin, beta A		−59.4
**NPNT**		nephronectin		−132.6
**SULT1B1**		sulfotransferase family, cytosolic,1B, member 1		−179.3
**SULT1E1**		sulfotransferase family 1E, estrogen-preferring, member 1		−307.6
***Syndecan-1 silencing***				
**LOC646891(SDCCAG3)**		similar to serologically defined colon cancer antig. 3		3.6
**SUSD3**		sushi domain containing	2.7	
**PCDHB17**		protocadherin beta 17 pseudogene	2.5	
**MARCH11**	membrane-associated ring finger (C3HC4) 11		2.4	
**FABP2**		fatty acid binding protein 2, intestinal	2.2	
**INSL5**		insulin-like 5	−2.0	
**DUSP19**		dual specificity phosphatase 19	−2.2	
**TMEM100**		transmembrane protein 100	−2.3	
**TTC32**		tetratricopeptide repeat domain 32	−2.5	
**UGT2B4**		UDP glucuronosyltransferase 2 family, polypepti B4	−2.5	
**LRRFIP1**		leucine rich repeat (in FLII) interacting protein 1	−2,7	
**FBLN5**		fibulin 5	−3.4	
**CYP4Z2P**		cytochrome P450, family 4, subfamily Z, polypeptide 2 pseudogene	−4.1	

FC represents fold changes at q≤0.05 of a gene following syndecan-1 modulation compared to cells transfected with the corresponding vector control.

**Figure 2:**
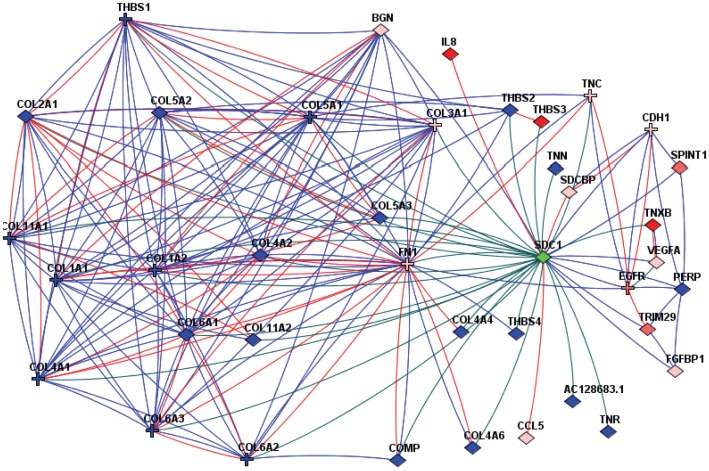
Most highly altered genes and their functional coupling to syndecan-1 according to the FunCoup network. The network view and layout was generated using the interface of FunCoup web site http://funcoup.sbc.su.se/. Red filled symbols denote genes in top 100 differentially expressed lists; pink symbols denote genes in top 900 differentially expressed lists. Blue filled symbols indicate other interactors of syndecan-1 in the analyzed network, beyond differentially expressed lists. Crosses correspond to genes with reported somatic point mutations by The Cancer Genome Atlas dataset (TCGA, 2008) [108]. Interactions are shown by lines: red lines = physical protein−protein interactions; blue lines = links established via co-expression in multiple datasets; green lines = links from KEGG pathways.

To show the differentially expressed genes which might be direct binding partners of syndecan-1 according to the currently available literature data, a global network of functional coupling (FunCoup), merged for higher coverage with curated resources KEGG and CORUM was used. The resulting network associates syndecan-1 expression with several cellular, but mostly extracellular compounds such as collagens, tenascin, fibronectin, VEGFA, IL8, syndecan-binding protein, etc. (Figure 2). Thus, the list of differentially expressed genes show several interesting patterns that either relate to previous knowledge or could motivate further investigation. However, a systematic approach was needed (i) to allow functional generalization; (ii) to convey confidence of observations; (iii) to help interpretation of differential expression of un-annotated genes. Such outcome is commonly provided by methods of gene set enrichment and network analysis.

### Validation of Microarray Data on RNA and Protein Level

**Figure 3:**
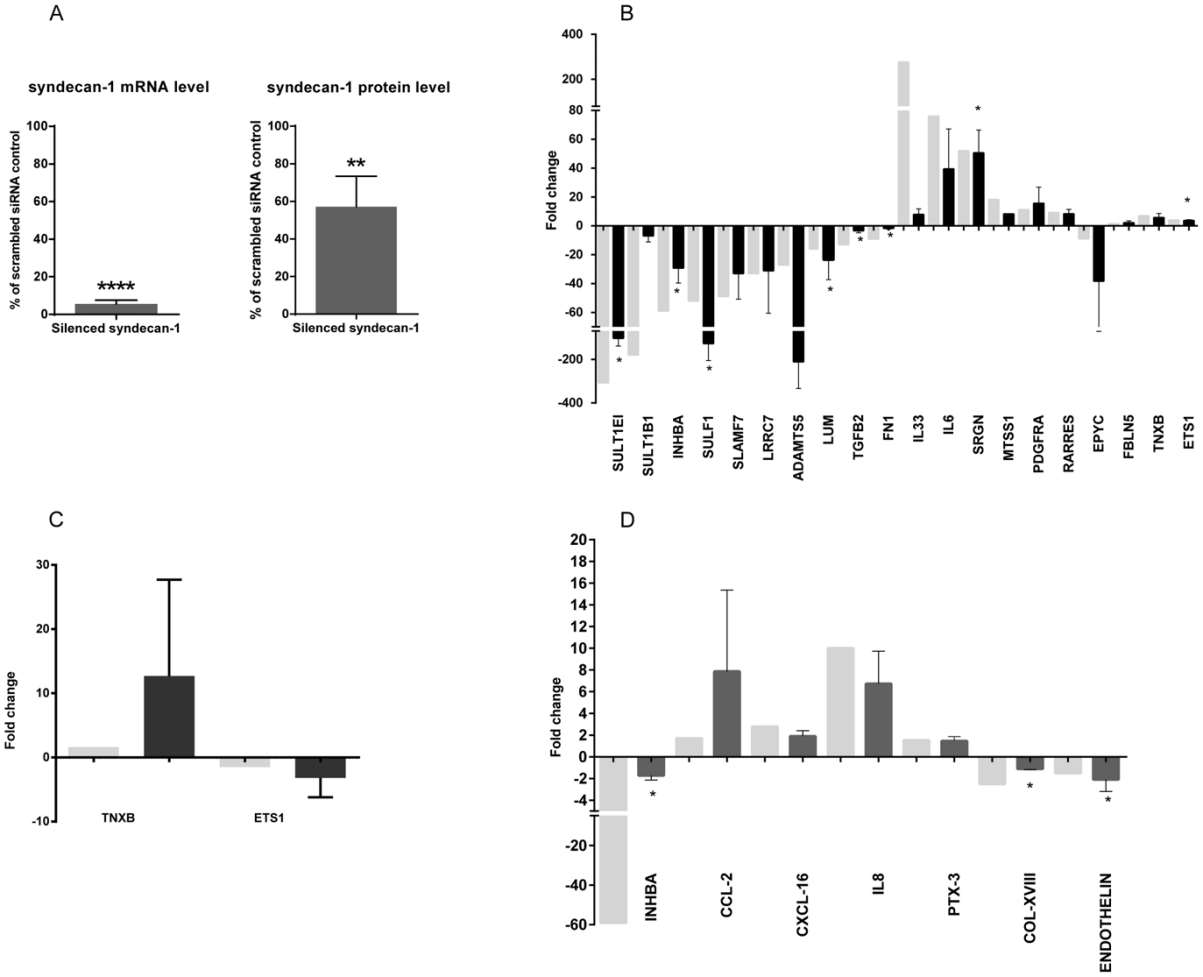
Validation of syndecan-1 silencing (A) and microarray data (B–D). (**A**) Succesful silencing of syndecan-1 was confirmed by RT-PCR and flow cytometry. The level of syndecan-1 mRNA (left column) and protein (right column) after silencing compared to cells treated with negative/scrambled control, 24 hours after silencing. The silencing is highly significant (p<0.0001 for mRNA and p<0.01 for protein ) as calculated by a one-sided t-test (n = 3). Selected transcripts deregulated in microarray were analyzed by RT-PCR for syndecan-1 overexpression (**B**) and silencing (**C**) and/or by proteome profile array (**D**) for syndecan-1 overexpression. mRNA or protein level of cells with modulated syndecan-1 was compared to their specific control (vector for overexpressed and negative-scrambled for silenced). Results are given in fold changes (FC). Light gray columns represent the values obtained by microarray, dark gray columns correspond to the results from RT-PCR or from proteome profiler array. Each value represents an average of fold-changes of three independent experiments, error bars represent standard deviation (SD); statistically significant changes: *p<0.05, ** p<0.01, **** p<0.0001 for differential expression of transcripts in syndecan-1 modulated cells compared to the corresponding controls.

The extent of syndecan-1 silencing was measured 24 hours after transfection and it corresponded to a 90–95% knockdown of the target mRNA and 43% knockdown of the protein compared to the scrambled control (Figure 3A). Syndecan-1 overexpression resulted in a 7 fold increase of mRNA level, and 2.5 fold increase of protein level, respectively [14].

For validation of microarray data, a sub-set of differentially expressed genes were selected corresponding to the highest FC and those which were DE in both silenced and overexpressed samples. The direction of changes in gene expression was concordant with the microarray data (Figure 3B–3C), although the obtained values differed in some cases from the array data, in line with previously reported microarray studies [33]. Significantly altered genes correspond to SULT1EI, INHBA, SULF1, LUM, TGFB2, SRGN, COL-XVIII, endothelin and ETS-1. Expression of selected genes was further validated on protein level. Seven out of 11 proteins showed concordant changes with the microarray analysis (Figure 3D).

### Functional Characterization of Genes Influenced by Syndecan-1 Overexpression

**Figure 4:**
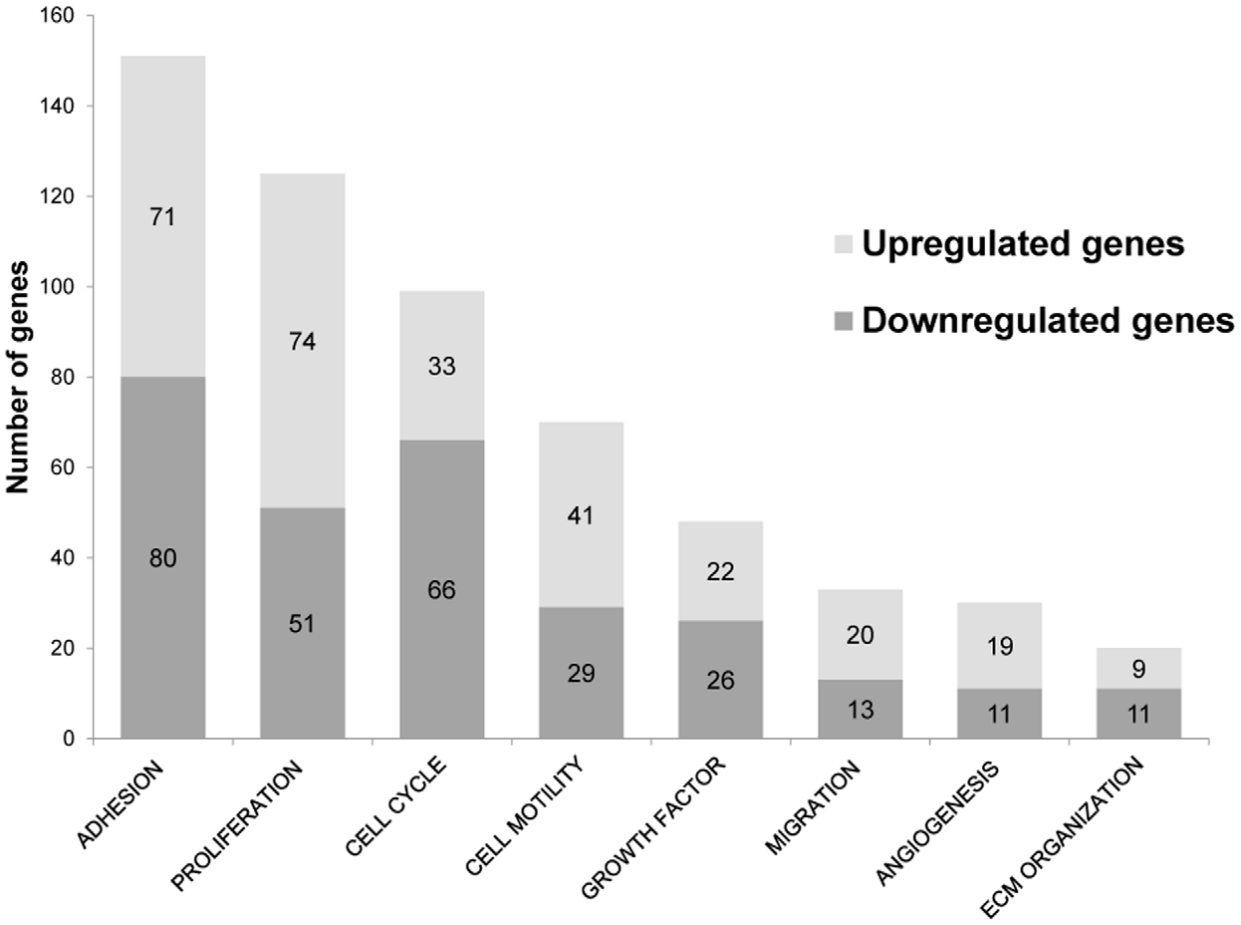
Differentially expressed genes following syndecan-1 overexpression grouped by functional categories according to Gene Ontology. Each differentially expressed gene (FC≥1.5 or FC ≤−1.5, q≤0.05 using OCplus test from R package) was assigned to functional categories using Gene Ontology database. Genes from each category were counted and plotted on the graph. Vertical axis represents the number of differentially expressed genes and each column represents a GO category.

The differentially expressed genes in syndecan-1 overexpressing cells were placed into functional categories according to their biological functions using Gene Ontology (GO) terms. The most frequently altered categories corresponded to cell adhesion with 151 genes, followed by proliferation (125 genes), motility (70 genes) and cell migration (33 genes) (Figure 4). A number of cytokines were differentially expressed, comprising several chemokines (CXCL1, CXCL16, CCL2 and CCL19), which were all upregulated. Seven out of 51 interleukins and five out of 41 interleukin receptors were upregulated and none of them were downregulated. Furthermore, IL33, IL6 and IL8 were more than 10 fold enhanced (Table S4).

The analysis strongly suggests that syndecan-1 affects cell proliferation: from 783 proliferation related genes on the chip, 51 were downregulated and 74 were upregulated (gene set enrichment p<0.001 by chi-square test, GraphPad, Prism). Within these, expression of 19 out of 150 growth factors (p = 0.048) and 14 out of 31 growth factor receptors (p<0.001) was also significantly altered (Table S4). The regulation of TGFβ family members and their receptors showed a more complex pattern: 6 ligands and 4 receptors were downregulated while three others were upregulated. Expression of EGF and its receptors as well as members of the VEGF family were also enhanced. PDGFC was three times downregulated while its receptors (PDGFRA, PDGFRB and PDGFRL) were upregulated. In the FGF family, FGF18 was slightly downregulated, while out of the four FGF receptors expression of FGFR2 was more than 6 times enhanced.

**Figure 5:**
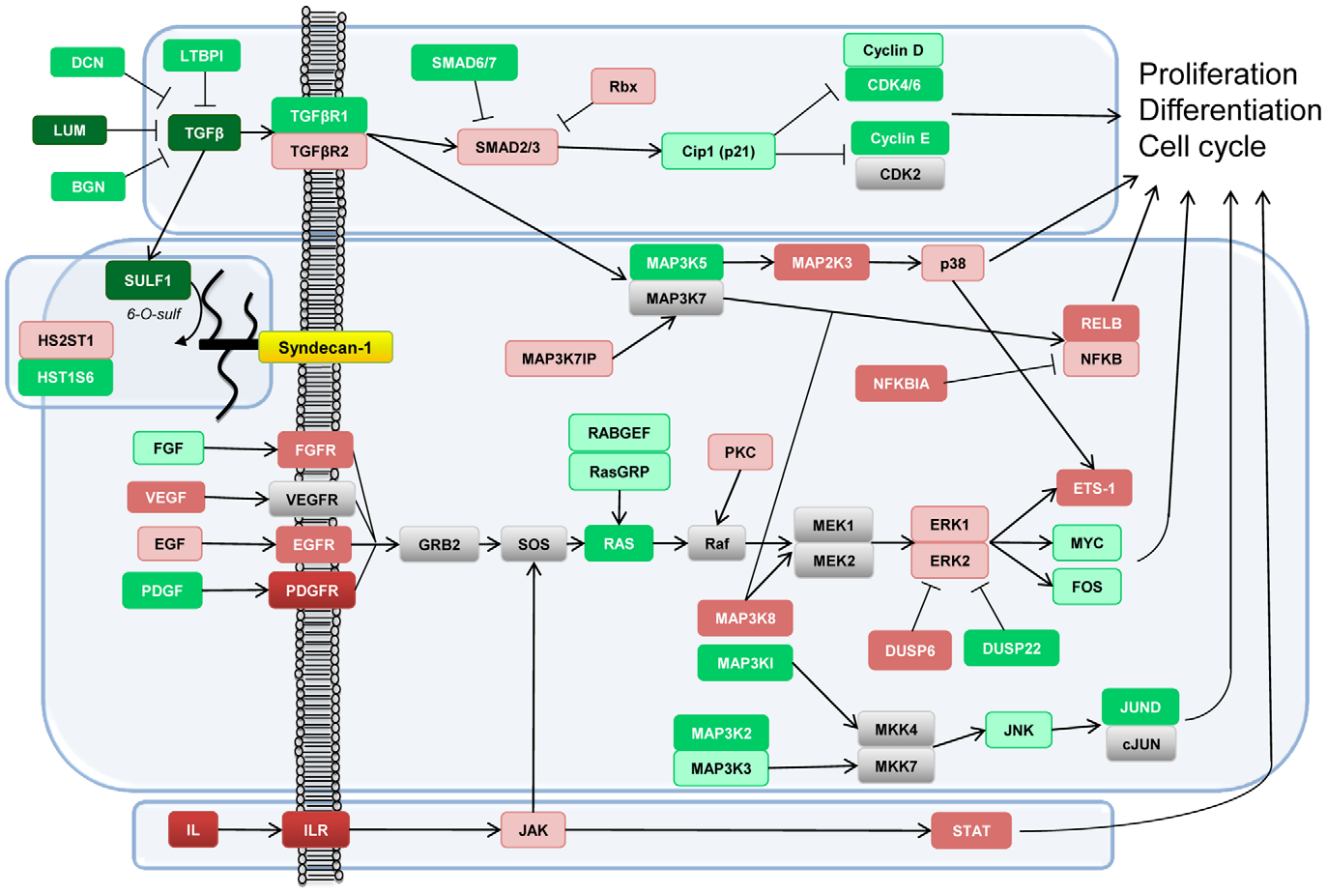
Schematic representation of genes and signaling pathways affected by syndecan-1 overexpression based on KEGG pathways. The figure summarizes the most important findings related to syndecan-1 overexpression. Green, red and gray boxes respectively show downregulated, upregulated and unaffected genes by syndecan-1 overexpression. Color intensity is proportional with fold change (FC): dark red and dark green correspond to a FC≥9, red and green correspond to a FC<9 and FC≥2, light red and green correspond to a FC<2 and FC≥1.5. Syndecan-1 acts on several growth-factor related pathways at multiple levels as illustrated by the TGF-beta pathway. The elements of JAK-STAT pathway are enhanced. The expression of growth factor receptors is mainly overexpressed. The MAPK cascade is perturbed at different points, receiving both direct and indirect signals, collectively leading to alterations of various transcription factors and cellular responses such as proliferation and cell-cycle regulation.

Furthermore, 99 genes involved in cell cycle regulation were differentially expressed, the majority being downregulated, especially those which drive the G1/S phases (e.g. cyclin E and the cyclinD/CDK6 complex). Downstream from the growth factors, 7 MAPK, MAPKK and MAPKKK genes were upregulated and 4 were downregulated. Their effectors, two FOS genes, JunD, and MYC genes were also downregulated. In addition, the expression of a number of MAPK inhibitors and dual specificity phosphatases (DUSP6, 8 and 22) was affected. Two Janus kinases (JAK1 and JAK2) as well as signal transducers and activators of transcription (STAT2, STAT5A and STAT6) were enhanced. The differentially expressed genes and potential pathways responsive to syndecan-1 overexpression are summarized in Figure 5. Proliferation and/or cell cycle progression related pathways were incorporated in the figure based on the KEGG database. Relevance of the pathway was validated by network enrichment analysis. Individual genes in the pathway were significantly linked to differentially expressed lists (either FL2E or SI2NS) taken as groups, requiring at least 3 network links between a pathway gene and list members (NEA p-value<0.05; FDR<0.1). Few genes were enriched in network connections to SI2NS differentially expressed list: GRB2, IL8, JAK1, JAK2 and MAP3K3 and all of these were also linked to the FL2E list. The latter observation suggests feedback loops of both syndecan-1 overexpression (mostly through IL8, IL6, MAPK1, MAPK3, BMP2K, CCL2, STAT5A) and syndecan-1down-regulation (through FRYL, MAPK3, PRKAA1, WEE1).

Syndecan-1 over-expression was followed by downregulation of extracellular small leucine reach repeat proteoglycans such as biglycan, epiphycan, decorin and lumican. Among the transmembrane and intracellular proteoglycans syndecan-2, serglycin and two members of glypican family were also differentially expressed (Table S4).

Enzymes involved in proteoglycan metabolism such as aggrecanase (ADAMTS5), membrane-associated matrix metalloproteases (MMP-15, -16 -24, ADAM-2, -15 and -23) and the tissue inhibitor of metalloproteinase 3 (TIMP-3) were significantly affected. Furthermore, expression of enzymes of importance for heparan sulfate fine structure was highly influenced: HS-2-O-sulfotransferase-1 was slightly upregulated, HS-6-O-sulfotransferase-1 was downregulated, SULF1, one of the genes responsible for the removal of 6-O-sulfate groups was in turn 52 fold downregulated, as well as other lysosomal sulfatases (ARSA, ARSJ and SGSH) (Table S4).

### Cellular and Molecular Functions Influenced by Syndecan-1 According to Ingenuity Pathway Analysis (IPA)

**Figure 6:**
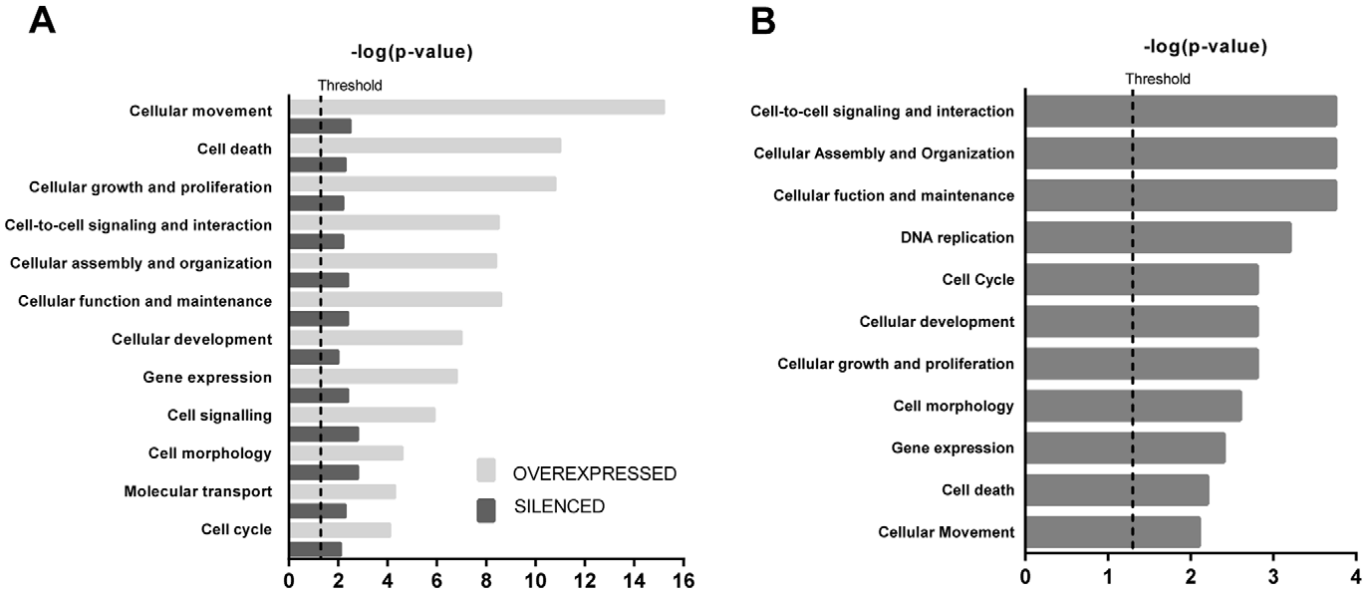
Most significantly altered cellular and molecular functions revealed by Ingenuity Pathway Analysis (IPA). (**A**) Functions significantly associated to syndecan-1 overexpression or silencing. Light gray bars show the gene set for syndecan-1 overexpression, dark gray bars show the gene set for syndecan-1 silencing. (**B**) Functions altered concomitantly by both overexpression and silencing. Molecules from both datasets that met the FC≥1.5 or FC≤−1.5 and q≤0.05 cutoff criteria were considered for the analysis. Right-tailed Fisher’s exact test was used to calculate a p-value. Bars represent the logarithmic values of the significance level (p), the dashed line corresponds to the threshold of p = 0.05.

Cellular movement, cell death, cellular growth and proliferation, cellular signaling, development and cell cycle were among the most affected (Figure 6A). The same functions were also significantly affected when the 14 genes concordantly altered by both syndecan-1 overexpression and silencing were uploaded to IPA, although the level of significance was slightly different (Figure 6B).

**Figure 7:**
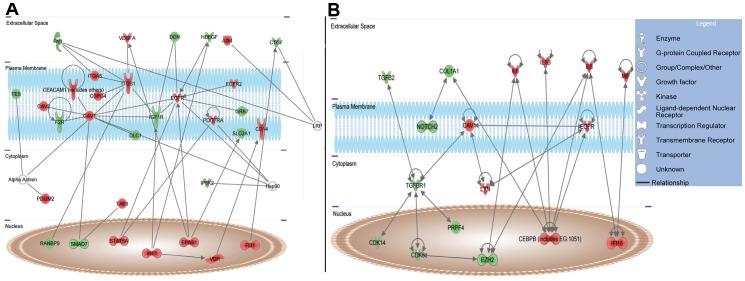
Networks related to proliferation (A) and cell-cycle regulation (B) according to Ingenuity Pathway (IPA). Differentially expressed genes which by functional analysis were associated to (**A**) proliferation or (**B**) cell cycle analysis were uploaded into IPA. Genes were overlaid onto a global molecular network developed from information contained in the Ingenuity® Knowledge Base. The obtained networks were algorithmically generated based on the connectivity of differentially expressed genes. The networks also reveal the relationship between different genes and their subcellular distribution according to IPA score from published data. Red symbols denote upregulated genes and green symbols denote downregulated genes.

The most significant networks generated from these data comprised genes with functions in inflammatory responses, cancer, cellular growth and proliferation, cellular development and gene expression (Figures S2 A and B). We re-analysed the dataset with overexpressed syndecan-1 focusing on two functional categories “Cellular growth and proliferation” and “Cell cycle”. The networks generated in this way tend to converge to TGFβ and EGFR (Figure 7). These networks supported our finding that modulation of syndecan-1 affects the process of cell proliferation and cell cycle at different levels comprising not only growth factors and cell surface receptors but also downstream kinases.

**Figure 8:**
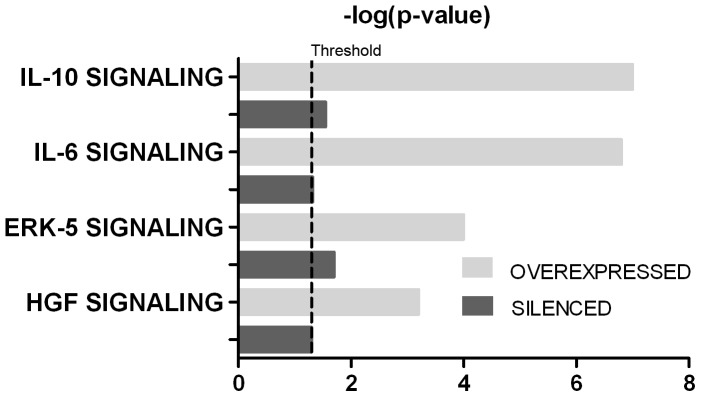
Common pathways significantly affected by both syndecan-1 overexpression and silencing, revealed by Ingenuity Pathway (IPA). Canonical pathway analysis identified the pathways from the IPA library that were most significant to the data set. Molecules from the data set that met the cutoff of FC≥1.5 or FC≤−1.5 and q≤0.05 and were associated with a canonical pathway in the Ingenuity Knowledge Base were considered for the analysis. The significance of the association between the data set and the canonical pathway was measured by Fisher’s exact test. Grey columns denote syndecan-1 overexpression and black columns denote syndecan-1 silencing. Bars represent the logarithmic values of the significance level (p), the dashed line represents the threshold of p = 0.05.

Five pathways were significantly altered by both syndecan-1 overexpression and silencing: two interleukin pathways (IL10 and IL6), the HGF pathway and the ERK5 and ERK/MAPK signaling pathways (Figure 8).

### Network Enrichment Analysis Over Known Functional Gene Sets

Genes were ranked by significance of differential expression, with a cut-off at 100 or 900 genes (q-value <0.05) (Table S5). The complete lists of differentially expressed genes with q-value<0.05 contained in total 2,539 genes. Functional relations between these lists (AGS) and various functional categories (FGS) were analyzed. The distribution of q-values is shown in Figure S1.

From more than 1,600 pathways analyzed, 939 were significantly altered in syndecan-1 overexpressing cells and 234 in syndecan-1 silenced cells. This large number can be expected given the extent of transcriptome alterations in our experiments and the fact that many of the FGSs overlapped and/or were largely synonymous (e.g. BioCarta, KEGG, and Reactome versions of same pathways). We further describe our observations presenting pairs of different AGS and FGSs related to each other (Table S5).

The most enriched pathways in syndecan-1 overexpressing cells were those associated with focal adhesion, EGF-receptor and ECM-receptor interaction pathways, with a NEA Z-score around 30 (top 900 gene set). Syndecan-mediated signaling events, glypican-network and HGF, PDGF, MAPK-related pathways were also among the most enriched pathways. Silencing of syndecan-1 highly altered several cell-cycle related pathways along with several cancer-related functional gene sets.

**Figure 9:**
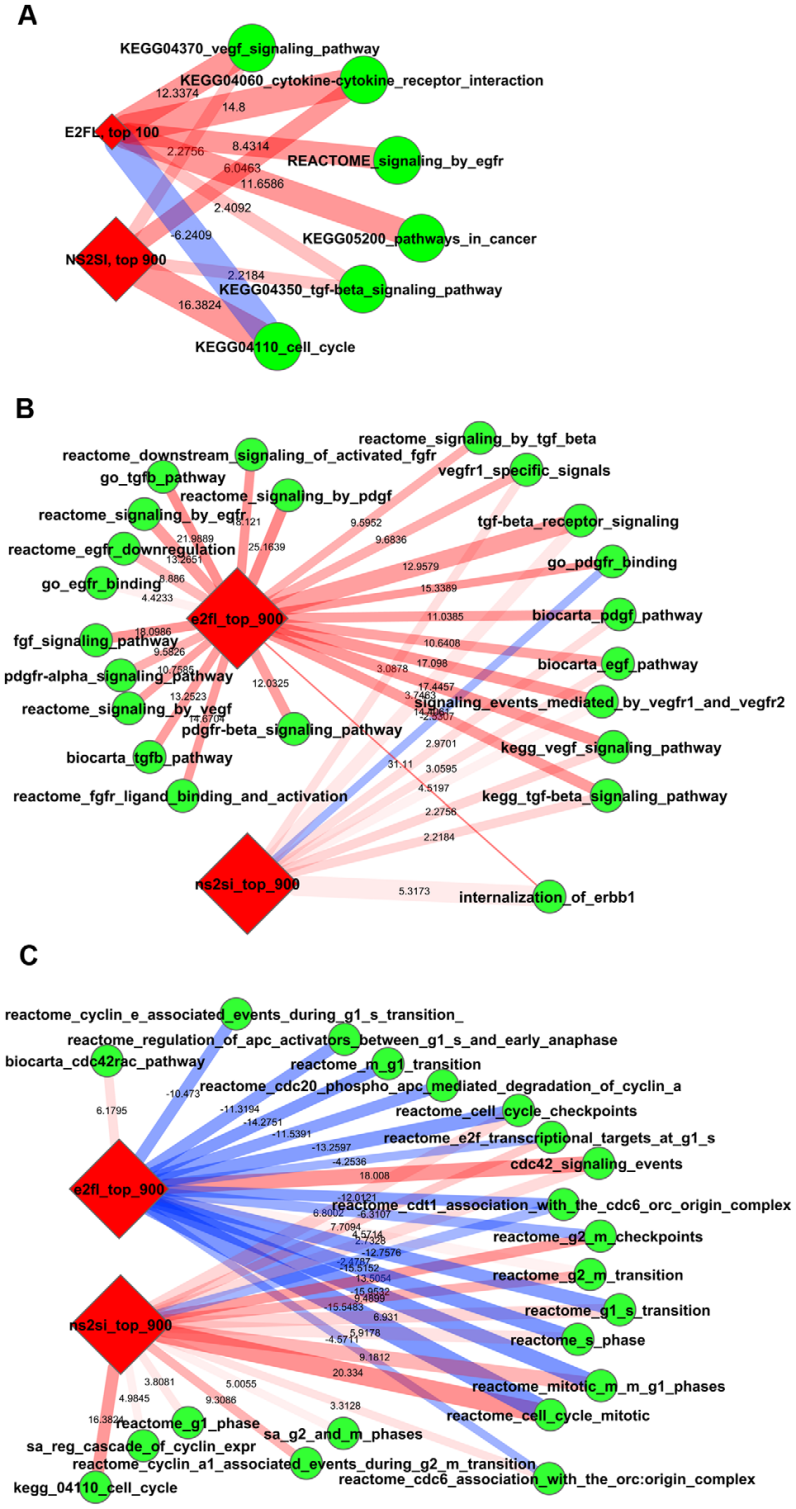
Pathways influenced by both syndecan-1 overexpression and silencing, revealed by network enrichment (NEA) analysis. Altered KEGG pathways (**A**); pathways determining signaling and growth factor activity (**B**); pathways related to particular phases of the cell cycle (**C**). Diamonds represent differentially expressed genes (E2FL denotes cells transfected with full-length syndecan-1 vs. cells transfected with empty vector; NS2SI denotes cells silenced for syndecan-1vs scrambled control); circles represent pathways from different databases (FGS). Red lines denote enriched pathways, blue lines denote depleted pathways. Line width is proportional with the number of individual gene**-**gene network links between two gene sets (linear scales from *min* 7 to *max* 3,995). Line opacity represents confidence (FDR<0.1 for every shown link). Numbers at lines correspond to Z-scores from network enrichment analysis of differentially expressed genes as groups versus genes of FGS as groups. The picture was generated using the stand-alone network software Cytoscape [109] using network edges and nodes from the custom NEA software.

The results further expanded the findings from GSEA and IPA analysis: many growth-factor, cytokine and cell cycle related pathways were altered following both syndecan-1 overexpression and silencing (Figure 9A). Cell-cycle pathways were enriched when syndecan-1 was silenced. Interestingly, many of the same pathways were depleted whereas the cdc42-related were enriched when syndecan-1 was overexpressed (Figure 9B). Using detailed sub-pathways, we could relate the changes to different phases of cell cycle, among which pathways regulating G1S and G2M checkpoints were the most altered. EGF, TGF, VEGF and PDGF pathways (Figure 9C) and several MAPK/ERK/JNK and JAK/STAT pathways were enriched both when syndecan-1 was overexpressed and silenced.

## Discussion

In order to interpret alterations generated by syndecan-1 in mesothelioma cells, we combined traditional strategies of gene expression analysis with a novel network enrichment analysis, which takes into account functional coupling in gene networks [29], [34].

Syndecan-1 overexpression profoundly affected a number of cytokines, growth factors and their receptors, extracellular matrix proteins, and genes regulating the sulfation pattern of heparan sulfate, hence altering many important signaling pathways. The observed pattern of differential expression could be coupled to functional categories by the Funcoup-based network [29], which includes literature data of direct binding partners of syndecan-1 (Figure 2). Most members of this network lay downstream of syndecan-1 and can be directly or indirectly regulated by the proteoglycan itself. Interestingly, genes that encode interactors of syndecan-1 not yet shown to be under syndecan-1 control, were perturbed as well, which suggests feed-back loops in syndecan-1 signaling. Further associations were obtained with the network enrichment analysis that summarizes functional responses over hundreds of differentially expressed genes and multiple pathways.

Many important components of signaling pathways employ molecular mechanisms other than transcription regulation. As Network Enrichment Analysis (NEA) looks at differentially expressed (DE) genes and their network relations to any functional gene set (FGS) members, it could detect FGSs where only few members are regulated at the transcription level (such as pathways involving MAPKs), allowing us to look beyond experimentally detected transcriptome alterations. The employed data integration network combined most known functional relations between genes and proteins. It elucidated relations of DE genes to functional categories via e.g. peptide chain modification, protein phosphorylation, miRNA regulation etc. The functional coupling enabled us to observe links between DE genes and pathways that determine functional responses or regulatory loops. The IPA approach is more limited to transcriptome changes as it performs gene set enrichment analysis on smaller hypothetical network modules of DE genes rather than on the whole network. In addition, for FunCoup-based interaction network it was also possible to trace particular network links back to the source of evidence.

This broader systemic approach indicates that syndecan-1 plays a central role in most functions considered hallmarks of cancer, including adhesion, migration, proliferation, invasion, cell cycle regulation, cell death and angiogenesis. Since adhesion, motility and migration-related functions have been extensively studied [14], [35], [36], [37], [38], in the present paper we focus on features related to tumor proliferation and growth.

Our previous study showed that syndecan-1 overexpression hampers proliferation in mesothelioma cells [13]. Interestingly, in this cell line, silencing of the same proteoglycan had a similar effect. While the inhibition of cell growth was accompanied by a prolonged S phase due to syndecan-1 overexpression [13], silencing showed accumulation of cells in G0/G1 phase with less cells in G2/M. Thus, we can presume that the mechanisms governing these effects might be different. The significant downregulation of cyclin E2 and cyclin D1-cdk4/6 complexes (Figure 5), key regulators of the G1 phase and G1/S transition, might partly explain the effects of syndecan-1 overexpression on proliferation. In parallel, cdk inhibitor p^21waf1/cip1^ was also inhibited. Interestingly, during overexpression of syndecan-1 many cell-cycle related pathways were depleted, i.e. the number of functional links between our dataset and the pathway in question was less than expected, whereas in silenced cells the same pathways were enriched comprising all phases of cell cycle (Figure 9C). The cdc42 related pathways were enriched regardless of the direction of syndecan-1 modulation, although the cdc42 gene itself was not differentially regulated.

The finding that both syndecan-1 overexpression and silencing suppressed proliferation may seem paradoxal. However, accumulating evidence suggest that syndecan-1 influences tumor growth and proliferation in a complex and tumor type specific manner [13], [39], [40], [41], [42], [43], [44]. In a recent study on HT1080 fibrosarcoma cell line syndecan-1 overexpression promoted proliferation along with the activation of genes driving the G1S transition [45], whereas in B6FS fibrosarcoma cell line the opposite effect was found [13]. The effect of syndecan-1 may partly depend on its endogenous level in the specific cell type studied as well as the ratio between membrane bound and shed syndecan, competing for ligand binding as shown in a breast cancer cell line, where overexpression of wild type syndecan-1 increased proliferation, but overexpression of constitutively shed syndecan-1 inhibited it [46]. The current study suggests that syndecan-1 is important in maintaining a delicate balance, regulating cell proliferation. Even a small perturbation of this balance may be followed by major changes in the behavior of cells. It is possible that an optimal concentration of syndecan-1 is necessary to induce some pathways while altered levels will be detrimental. Such a “bell-shaped” dose-response curve with a maximal response at a narrow concentration range is a pattern commonly found in heparan sulfate- or growth factor-mediated signaling [47], [48], [49], [50].

Modulation of syndecan-1 expression significantly enriched the cytokine-cytokine receptor interaction pathway and pathways where ILs were involved. In line with this, a group of pro-inflammatory ILs was highly upregulated due to syndecan-1 overexpression even though the baseline level was almost undetectable. Proliferation largely depends on growth factors and cytokines [51], [52], [53] and there is a delicate interplay between these two. Interestingly, some of the interleukins regulated by syndecan-1 in our study, such as IL8 acts also as an autocrine growth factor [54] whereas IL6 induces expression of VEGF in malignant mesothelioma [55]. Heparan sulfate can also initiate an inflammatory process through sequestration of cytokines in ECM [56]. By binding cytokines and presenting them to their receptors, syndecans regulate cytokine responses and thereby control inflammatory responses and cell proliferation [57], [58].

Growth factor receptors require syndecans as co-receptors for signaling (for review see [59]). In mesothelioma cells syndecan-1 influenced major growth factor pathways and many of these pathways were altered at multiple levels. Syndecan-1 overexpression was followed by downregulation of PDGF and FGF family members, while their receptors were upregulated. Simultaneously expression of both EGF and EGFR was enhanced. This is particularly interesting in the light of our previous report [60] where exposure of mesothelioma cells to EGF and IGF-1 inhibited expression of syndecan-1 and **-**2. Although HGF was not affected itself, we observed that both syndecan-1 silencing and overexpression significantly altered the HGF signaling pathway. This is in line with previous data showing that syndecan-1 strongly promotes HGF-induced signaling through MET, the tyrosine-kinase receptor for HGF, resulting in enhanced activation of signaling pathways involved in the control of cell proliferation and survival [61]. Our data suggest that these effects are not limited to cell-surface receptors but also influence their downstream effectors. Thus, modulation of growth factors and growth factor receptors was accompanied by a deregulation of ERK/MAPK, JNK and p38/MAPK pathways. Several elements of the kinase cascade were apparently contra regulated, and downstream of these, transcription factors like MYC, FOS, JNK and JUN were all downregulated. Interestingly, ETS-1 was upregulated due to syndecan-1 overexpression and inhibited due to syndecan-1 silencing. ETS-1 is a proto-oncogene which correlates to the prognosis in several tumors [62], [63], [64], [65]. It might be an important target of syndecan-1, connecting the different findings in our study, since it is known that ETS protein stimulates TGFβR promoter activity [66], regulates several cytokines, chemokines [67], MMPs [68], [69] and has a role in cell cycle regulation [70], [71].

Both TGFβ and TGFβR1 were highly downregulated upon syndecan-1 overexpression, while the downstream Smad3 was upregulated. Recently it was found that syndecan-1 can increase the level of phosphorylated Smad2 after TGFβ stimulation [72]. TGFβ induction initially hampers the proliferation of epithelial cancer and induces apoptosis. However, tumorigenesis may alter TGFβ signaling pathway to convert TGFβ from a tumor suppressor to a promoter of cell growth, invasion and metastasis and can have a role in enabling cancer cells to acquire epithelial-mesenchymal transition (EMT) [73]. TGFβ may enhance the growth of mesenchymal tumors and the reduction of TGFβ level in mesothelioma cells results in inhibition of tumor growth both *in vitro* and *in vivo* [74], [75]. On the other hand, when given to mesothelioma cells, TGFβ2 delayed the nuclear transport of syndecan-1 in parallel with an antiproliferative effect [60]. It has also been shown that syndecan-1 can function as a negative regulator of TGFβ signaling [76], which is in line with our present results. In our experimental settings downregulation of TGFβ in response to syndecan-1 overexpression is associated with an inhibition of proliferation. These data suggest that syndecan-1 is a powerful suppressor of the TGFβ mediated signaling, which warrants further investigations.

Literature data also suggest a connection between sulfatase 1 (SULF1) and TGFβ, SULF1 being a TGFβ responsive gene [77], [78]. In our dataset both TGFβ and SULF1 were highly downregulated as a result of syndecan-1 overexpression. SULF1 is one of the two enzymes responsible for the selective removal of the 6-O-sulfate groups from heparan sulfate chains Other enzymes responsible for heparan sulfate chain synthesis and sulfation were moderately altered (Table S4). Since the growth factor binding affinity of syndecan-1depends on the fine structure and particularly the sulfation of the heparan sulfate chains, downregulation of SULF1 might be one way by which syndecan-1 regulates cell growth, by modulating its own growth factor binding properties [79]. SULF1 has a dual role in enhancing or inhibiting different growth factor signaling pathways, thereby contributing to the modulation of proliferation. In accordance with the requirement of sulfated heparan sulfate chains for growth factor-growth factor receptor binding, SULF1 inhibits the activity of FGF [80], [81], [82] and also attenuates the activation of HB-EGF [83] and both ERK-MAP kinases and HGF mediated AKT signaling [84], [85]. SULF1 is on the other hand a known promoter of WNT signaling [86], [87] and there are evidences that it also activates other pathways, like BMP/Noggin signaling [88].

Previously it was assumed that SULF1 has a tumor suppressor role, and it is downregulated in many tumor types [89], [90]. However, in malignant mesothelioma and a wide range of other tumors [89] SULF1 is clearly overexpressed [91]. High SULF1 expression was associated with poor prognosis in adenocarcinoma [92], and silencing of this enzyme inhibited proliferation of pancreatic cancer cells [93]. It was suggested that cancers driven by WNT-1 signaling would likely be enhanced by SULF1, whereas others, where FGF2 or HGF signaling is the more significant driving mechanism, are inhibited [90], [93]. Our findings seem to fit in this hypothesis: in mesothelioma cells the massive downregulation of SULF1 correlates with a growth inhibition. We can hypothesize that in our experimental settings SULF1 downregulation can contribute to inhibition of proliferation, given the fact that the level of SULF1 was found elevated in this tumor compared to the normal mesothelium and there are evidences that Wnt pathway is also altered [94], [95], [96]. SULF1 can possibly also modulate many of the syndecan-1 related effects seen in our study, where a 3 fold overexpression of syndecan-1 was followed by massive deregulation of a high number of genes. This is the first report showing that syndecan-1 regulates the expression of SULF1, however, the functional significance of these findings necessitates further investigations.

Syndecan-1 overexpression also affects the expression of structurally related molecules such as other proteoglycans. Syndecan-2 was downregulated, which is in line with our previous report where overexpression of syndecan-1 leads to a changed syndecan profile [13]. Furthermore, recent evidence also suggests that there is a cooperativity between these two syndecans [45]. Upregulation of glypican-3 upon syndecan-1 overexpression may contribute to the negative effects seen on proliferation and shown to be proapoptotic in both breast cancer and mesothelioma cell lines [97]. Syndecan-1 driven upregulation of serglycin (the only constitutively intracellular proteoglycan) in mesothelioma cells can be an important new finding for cancer cell biology, as there are only a few reports linking serglycin to tumors, mostly to multiple myeloma [98] and nasopharyngeal carcinoma cells [99]. The sulfation pattern seems to be important for this proteoglycan in its role in cancer and the perturbations of the enzymes responsible for GAG sulfation may act also at this level. Serglycin is also involved in retention of proteases [100]. The ectodomain of syndecan-1 is released by the action of proteolytic cleavage, including mainly metalloproteases. Tissue inhibitor of metalloproteinase TIMP-3 has been shown to effectively block shedding of syndecan-1 and -4 [101], and it binds to sulfated GAGs, enabling interaction with the syndecans as well as with matrix proteoglycans [102], [103], [104]. Here we show that TIMP-3 can be in turn downregulated by syndecan-1. These results indicate that syndecan-1 modulation also may interfere with syndecan-1 shedding, a conclusion supported by a very recent concomitant study [105]. Thus syndecan-1 can affect the growth-factor gradient and thereby the availability of mitogens in the neighborhood of the cells.

Comparison with other array based screenings on cells with altered syndecan-1 expression, reveals potentially interesting downstream targets of syndecan-1 comprising cell cycle regulators, cdc42, MAPK, p21 [46], [106] and ETS-1 [45]. Concordant changes between different cell types are however limited and the overall changes are dissimilar, suggesting context- or cell-type specific effects of syndecan-1.

Evaluation of syndecan-1 modulation at these different levels of complexity (GSEA, IPA and NEA) complement each other, giving a complex view on how syndecan-1 orchestrates different growth factors, converging at downstream pathways. The multitude of biological processes thus influenced motivates the designation of syndecan-1 as “tuner of transmembrane signaling” [107].

To our knowledge, this is the first report elucidating the various molecular mechanisms regulated by syndecan-1 on a systemic level. One limitation of this study is that we used only one cell line, which is sufficient for the construction of a general model for syndecan-1 dependent pathways, but the general applicability of these pathways warrants subsequent studies. We identified key components and pathways directly or indirectly affected by syndecan-1 by combining functional assays with advanced bioinformatics. The observed deregulations include both increased and decreased expression of genes, having either stimulatory or inhibitory effects but ultimately leading to hampered proliferation. To address the individual contribution of the altered pathways, further functional studies are ongoing in our laboratory. A better understanding of the complex role of syndecan-1 and its molecular interactions in malignant mesothelioma may provide future possibilities to control tumor growth and proliferation.

## Supporting Information

Figure S1 **Distributions of false discovery rates (q) after differential expression analysis using paired t-test (R package OCplus**). Legends at each plot denote comparison of sample and respective control and the total number of microarray probes that passed the criterion q≤0.05. Full-length represents genes from cells transfected with full-length syndecan-1 (i.e. -syndecan-1 overexpressed), empty vector represents genes from cells transfected with empty vector (control construct for overexpressed syndecan-1), siRNA corresponds to genes from cells with silenced syndecan-1, negative-scrambled corresponds to cells transfected with scrambled control construct for silenced cells.
(TIF)

Figure S2 **The most significant networks generated using IPA analysis.** Networks were generated from genes altered by syndecan-1 overexpression (A) and silencing (B). The data set contains gene identifiers and corresponding expression values uploaded into the IPA application. A *q* cutoff of <0.05 was set to identify molecules whose expression was significantly differentially regulated. These molecules were overlaid onto a global molecular network in the Ingenuity Knowledge Base. Networks were then algorithmically generated based on their connectivity. Molecules are represented as nodes, and the biological relationship between two nodes is represented as an edge (line). All edges are supported by at least one reference from the literature, or from canonical information stored in the Ingenuity Knowledge Base. The intensity of the node color indicates the degree of up- (red) or downregulation (green). Nodes are displayed using various shapes that represent the functional class of the gene product. Edges are displayed with various labels that describe the nature of the relationship between the nodes. The associated functions correspond to Gene Expression, Inflammatory responses, Cancer for overexpression and Cellular development, Cell Growth and Proliferation, Gene Expression were associated to syndecan-1 silencing. For the graphical presentation the network analysis included the direct relationships only.
(TIF)

Table S1 **siRNA construct sequences used.**
(DOCX)

Table S2 **Primer sequences used for RT-PCR validation of differentially expressed genes and syndecan-1.**
(DOCX)

Table S3 **The rate of apoptotic cells in the syndecan-1 silenced cells compared to the scrambled siRNA control.** Apoptosis was measured by flow cytometry using Annexin-V-FITC and Propidium iodide (PI) staining, 24 and 48 hours after syndecan-1 silencing. The results are mean of three independent experiments ±SD. No significant changes were recorded in the rate of apoptotic cells.
(DOCX)

Table S4 **Selected functional categories affected by syndecan-1 overexpression.** Genes are grouped by using GO terms. FC = fold changes, all differences correspond to q≤0.05.
(DOCX)

Table S5 **Pairs of Altered Gene Sets (AGS) and Functional Gene Sets (FGS) according to their Z scores.** FL2E corresponds to a list of genes from the comparison of cells transfected with full-length syndecan-1 vs. cells transfected with empty vector; SI2NS corresponds to list of genes from the comparison of cells silenced for syndecan-1 vs scrambled control. Top 100 and 900 genes are listed in terms of q values. AGS represents lists of top 100 or 900 most significantly altered genes from the syndecan-1 modulated gene-set. FGS corresponds to the list of functional gene sets (pathways or GO categories) used for network enrichment analysis. SD: standard deviation; the standard Z-score was calculated based on the observed and expected link counts and their standard deviation, as described in materials and methods.
(XLSX)

## References

[1] Bernfield M, Gotte M, Park PW, Reizes O, Fitzgerald ML, et al (1999) Functions of cell surface heparan sulfate proteoglycans. Annu Rev Biochem 68: 729–777. 10872465 10.1146/annurev.biochem.68.1.729

[2] Liu W, Litwack ED, Stanley MJ, Langford JK, Lander AD, et al (1998) Heparan sulfate proteoglycans as adhesive and anti-invasive molecules. Syndecans and glypican have distinct functions. J Biol Chem 273: 22825–22832. 9712917 10.1074/jbc.273.35.22825

[3] Beauvais DM, Burbach BJ, Rapraeger AC (2004) The syndecan-1 ectodomain regulates alphavbeta3 integrin activity in human mammary carcinoma cells. J Cell Biol 167: 171–181. 15479743 10.1083/jcb.200404171 PMC2172512

[4] Beauvais DM, Rapraeger AC (2003) Syndecan-1-mediated cell spreading requires signaling by alphavbeta3 integrins in human breast carcinoma cells. Exp Cell Res 286: 219–232. 12749851 10.1016/s0014-4827(03)00126-5

[5] Lee H, Kim Y, Choi Y, Choi S, Hong E, et al (2011) Syndecan-2 cytoplasmic domain regulates colon cancer cell migration via interaction with syntenin-1. Biochem Biophys Res Commun 409: 148–153. 21569759 10.1016/j.bbrc.2011.04.135

[6] Lee JH, Park H, Chung H, Choi S, Kim Y, et al (2009) Syndecan-2 regulates the migratory potential of melanoma cells. J Biol Chem 284: 27167–27175. 19641225 10.1074/jbc.M109.034678 PMC2785644

[7] Purushothaman A, Uyama T, Kobayashi F, Yamada S, Sugahara K, et al (2010) Heparanase-enhanced shedding of syndecan-1 by myeloma cells promotes endothelial invasion and angiogenesis. Blood 115: 2449–2457. 20097882 10.1182/blood-2009-07-234757 PMC2845901

[8] Beauvais DM, Ell BJ, McWhorter AR, Rapraeger AC (2009) Syndecan-1 regulates alphavbeta3 and alphavbeta5 integrin activation during angiogenesis and is blocked by synstatin, a novel peptide inhibitor. J Exp Med 206: 691–705. 19255147 10.1084/jem.20081278 PMC2699122

[9] Elenius K, Jalkanen M (1994) Function of the syndecans–a family of cell surface proteoglycans. J Cell Sci 107 (Pt 11): 2975–2982. 10.1242/jcs.107.11.2975 7698997

[10] Tumova S, Woods A, Couchman JR (2000) Heparan sulfate proteoglycans on the cell surface: versatile coordinators of cellular functions. Int J Biochem Cell Biol 32: 269–288. 10716625 10.1016/s1357-2725(99)00116-8

[11] Choi Y, Chung H, Jung H, Couchman JR, Oh ES (2011) Syndecans as cell surface receptors: Unique structure equates with functional diversity. Matrix Biol 30: 93–99. 21062643 10.1016/j.matbio.2010.10.006

[12] Fears CY, Woods A (2006) The role of syndecans in disease and wound healing. Matrix Biol 25: 443–456. 16934444 10.1016/j.matbio.2006.07.003

[13] Zong F, Fthenou E, Castro J, Peterfia B, Kovalszky I, et al (2010) Effect of syndecan-1 overexpression on mesenchymal tumour cell proliferation with focus on different functional domains. Cell Prolif 43: 29–40. 19840029 10.1111/j.1365-2184.2009.00651.x PMC6496211

[14] Zong F, Fthenou E, Mundt F, Szatmari T, Kovalszky I, et al (2011) Specific syndecan-1 domains regulate mesenchymal tumor cell adhesion, motility and migration. PLoS One 6: e14816. 21731601 10.1371/journal.pone.0014816 PMC3121713

[15] Yang H, Testa JR, Carbone M (2008) Mesothelioma epidemiology, carcinogenesis, and pathogenesis. Curr Treat Options Oncol 9: 147–157. 18709470 10.1007/s11864-008-0067-z PMC2717086

[16] Dobra K, Andang M, Syrokou A, Karamanos NK, Hjerpe A (2000) Differentiation of mesothelioma cells is influenced by the expression of proteoglycans. Exp Cell Res 258: 12–22. 10912783 10.1006/excr.2000.4915

[17] Kumar-Singh S, Jacobs W, Dhaene K, Weyn B, Bogers J, et al (1998) Syndecan-1 expression in malignant mesothelioma: correlation with cell differentiation, WT1 expression, and clinical outcome. J Pathol 186: 300–305. 10211120 10.1002/(SICI)1096-9896(1998110)186:3<300::AID-PATH180>3.0.CO;2-Q

[18] Gulyas M, Hjerpe A (2003) Proteoglycans and WT1 as markers for distinguishing adenocarcinoma, epithelioid mesothelioma, and benign mesothelium. J Pathol 199: 479–487. 12635139 10.1002/path.1312

[19] Abatangelo L, Maglietta R, Distaso A, D’Addabbo A, Creanza TM, et al (2009) Comparative study of gene set enrichment methods. BMC Bioinformatics 10: 275. 19725948 10.1186/1471-2105-10-275 PMC2746222

[20] Alexeyenko A, Lee W, Pernemalm M, Guegan J, Dessen P, et al (2012) Network enrichment analysis: extension of gene-set enrichment analysis to gene networks. BMC Bioinformatics 13: 226. 22966941 10.1186/1471-2105-13-226 PMC3505158

[21] Ridley RC, Xiao H, Hata H, Woodliff J, Epstein J, et al (1993) Expression of syndecan regulates human myeloma plasma cell adhesion to type I collagen. Blood 81: 767–774. 8427968

[22] Takada Y, Shinkai F, Kondo S, Yamamoto S, Tsuboi H, et al (1994) Fluid shear stress increases the expression of thrombomodulin by cultured human endothelial cells. Biochem Biophys Res Commun 205: 1345–1352. 7802668 10.1006/bbrc.1994.2813

[23] Pawitan Y, Michiels S, Koscielny S, Gusnanto A, Ploner A (2005) False discovery rate, sensitivity and sample size for microarray studies. Bioinformatics 21: 3017–3024. 15840707 10.1093/bioinformatics/bti448

[24] Thomas S, Bonchev D (2010) A survey of current software for network analysis in molecular biology. Hum Genomics 4: 353–360. 20650822 10.1186/1479-7364-4-5-353 PMC3500165

[25] Berriz GF, King OD, Bryant B, Sander C, Roth FP (2003) Characterizing gene sets with FuncAssociate. Bioinformatics 19: 2502–2504. 14668247 10.1093/bioinformatics/btg363

[26] Hanahan D, Weinberg RA (2011) Hallmarks of cancer: the next generation. Cell 144: 646–674. 21376230 10.1016/j.cell.2011.02.013

[27] Hanahan D, Weinberg RA (2000) The hallmarks of cancer. Cell 100: 57–70. 10647931 10.1016/s0092-8674(00)81683-9

[28] Kanehisa M, Goto S, Furumichi M, Tanabe M, Hirakawa M (2010) KEGG for representation and analysis of molecular networks involving diseases and drugs. Nucleic Acids Res 38: D355–360. 19880382 10.1093/nar/gkp896 PMC2808910

[29] Alexeyenko A, Sonnhammer EL (2009) Global networks of functional coupling in eukaryotes from comprehensive data integration. Genome Res 19: 1107–1116. 19246318 10.1101/gr.087528.108 PMC2694487

[30] Ruepp A, Waegele B, Lechner M, Brauner B, Dunger-Kaltenbach I, et al (2010) CORUM: the comprehensive resource of mammalian protein complexes–2009. Nucleic Acids Res 38: D497–501. 19884131 10.1093/nar/gkp914 PMC2808912

[31] Staton CA, Kumar I, Reed MW, Brown NJ (2007) Neuropilins in physiological and pathological angiogenesis. J Pathol 212: 237–248. 17503412 10.1002/path.2182

[32] Pompeo E, Albonici L, Doldo E, Orlandi A, Manzari V, et al (2009) Placenta growth factor expression has prognostic value in malignant pleural mesothelioma. Ann Thorac Surg 88: 426–431. 19632388 10.1016/j.athoracsur.2009.04.038

[33] Morey JS, Ryan JC, Van Dolah FM (2006) Microarray validation: factors influencing correlation between oligonucleotide microarrays and real-time PCR. Biol Proced Online 8: 175–193. 17242735 10.1251/bpo126 PMC1779618

[34] Alexeyenko A, Schmitt T, Tjarnberg A, Guala D, Frings O, et al.. (2011) Comparative interactomics with Funcoup 2.0. Nucleic Acids Res. 10.1093/nar/gkr1062 PMC3245127 22110034

[35] Katz BZ (2010) Adhesion molecules–The lifelines of multiple myeloma cells. Semin Cancer Biol 20: 186–195. 20416379 10.1016/j.semcancer.2010.04.003

[36] Vuoriluoto K, Hognas G, Meller P, Lehti K, Ivaska J (2011) Syndecan-1 and -4 differentially regulate oncogenic K-ras dependent cell invasion into collagen through alpha2beta1 integrin and MT1-MMP. Matrix Biol 30: 207–217. 21414405 10.1016/j.matbio.2011.03.003

[37] Yang N, Mosher R, Seo S, Beebe D, Friedl A (2011) Syndecan-1 in breast cancer stroma fibroblasts regulates extracellular matrix fiber organization and carcinoma cell motility. Am J Pathol 178: 325–335. 21224069 10.1016/j.ajpath.2010.11.039 PMC3069862

[38] Stepp MA, Daley WP, Bernstein AM, Pal-Ghosh S, Tadvalkar G, et al (2010) Syndecan-1 regulates cell migration and fibronectin fibril assembly. Exp Cell Res 316: 2322–2339. 20580707 10.1016/j.yexcr.2010.05.020 PMC3141227

[39] Maeda T, Alexander CM, Friedl A (2004) Induction of syndecan-1 expression in stromal fibroblasts promotes proliferation of human breast cancer cells. Cancer Res 64: 612–621. 14744776 10.1158/0008-5472.can-03-2439

[40] Derksen PW, Keehnen RM, Evers LM, van Oers MH, Spaargaren M, et al (2002) Cell surface proteoglycan syndecan-1 mediates hepatocyte growth factor binding and promotes Met signaling in multiple myeloma. Blood 99: 1405–1410. 11830493 10.1182/blood.v99.4.1405

[41] Mali M, Elenius K, Miettinen HM, Jalkanen M (1993) Inhibition of basic fibroblast growth factor-induced growth promotion by overexpression of syndecan-1. J Biol Chem 268: 24215–24222. 8226969

[42] Sanderson RD, Yang Y (2008) Syndecan-1: a dynamic regulator of the myeloma microenvironment. Clin Exp Metastasis 25: 149–159. 18027090 10.1007/s10585-007-9125-3 PMC3633534

[43] Anttonen A, Heikkila P, Kajanti M, Jalkanen M, Joensuu H (2001) High syndecan-1 expression is associated with favourable outcome in squamous cell lung carcinoma treated with radical surgery. Lung Cancer 32: 297–305. 11390011 10.1016/s0169-5002(00)00230-0

[44] Lundin M, Nordling S, Lundin J, Isola J, Wiksten JP, et al (2005) Epithelial syndecan-1 expression is associated with stage and grade in colorectal cancer. Oncology 68: 306–313. 16020957 10.1159/000086969

[45] Peterfia B, Fule T, Baghy K, Szabadkai K, Fullar A, et al (2012) Syndecan-1 Enhances Proliferation, Migration and Metastasis of HT-1080 Cells in Cooperation with Syndecan-2. PLoS One 7: e39474. 22745764 10.1371/journal.pone.0039474 PMC3383727

[46] Nikolova V, Koo CY, Ibrahim SA, Wang Z, Spillmann D, et al (2009) Differential roles for membrane-bound and soluble syndecan-1 (CD138) in breast cancer progression. Carcinogenesis 30: 397–407. 19126645 10.1093/carcin/bgp001

[47] Rubin JB, Choi Y, Segal RA (2002) Cerebellar proteoglycans regulate sonic hedgehog responses during development. Development 129: 2223–2232. 11959830 10.1242/dev.129.9.2223

[48] Zhu H, Duchesne L, Rudland PS, Fernig DG (2010) The heparan sulfate co-receptor and the concentration of fibroblast growth factor-2 independently elicit different signalling patterns from the fibroblast growth factor receptor. Cell Commun Signal 8: 14. 20576134 10.1186/1478-811X-8-14 PMC2912315

[49] Schwall RH, Chang LY, Godowski PJ, Kahn DW, Hillan KJ, et al (1996) Heparin induces dimerization and confers proliferative activity onto the hepatocyte growth factor antagonists NK1 and NK2. J Cell Biol 133: 709–718. 8636243 10.1083/jcb.133.3.709 PMC2120823

[50] Rek A, Brandner B, Geretti E, Kungl AJ (2009) A biophysical insight into the RANTES-glycosaminoglycan interaction. Biochim Biophys Acta 1794: 577–582. 19455751 10.1016/j.bbapap.2009.01.001

[51] Vetvicka V, Vetvickova J (2011) Procathepsin D and cytokines influence the proliferation of lung cancer cells. Anticancer Res 31: 47–51. 21273579

[52] Evans C, Morrison I, Heriot AG, Bartlett JB, Finlayson C, et al (2006) The correlation between colorectal cancer rates of proliferation and apoptosis and systemic cytokine levels; plus their influence upon survival. Br J Cancer 94: 1412–1419. 16641913 10.1038/sj.bjc.6603104 PMC2361288

[53] Eiro N, Vizoso FJ (2012) Inflammation and cancer. World J Gastrointest Surg 4: 62–72. 22530080 10.4240/wjgs.v4.i3.62 PMC3332223

[54] Galffy G, Mohammed KA, Dowling PA, Nasreen N, Ward MJ, et al (1999) Interleukin 8: an autocrine growth factor for malignant mesothelioma. Cancer Res 59: 367–371. 9927048

[55] Adachi Y, Aoki C, Yoshio-Hoshino N, Takayama K, Curiel DT, et al (2006) Interleukin-6 induces both cell growth and VEGF production in malignant mesotheliomas. Int J Cancer 119: 1303–1311. 16642474 10.1002/ijc.22006

[56] Gill S, Wight TN, Frevert CW (2010) Proteoglycans: key regulators of pulmonary inflammation and the innate immune response to lung infection. Anat Rec (Hoboken) 293: 968–981. 20503391 10.1002/ar.21094 PMC4121077

[57] Gotte M (2003) Syndecans in inflammation. Faseb J 17: 575–591. 12665470 10.1096/fj.02-0739rev

[58] Gotte M, Echtermeyer F (2003) Syndecan-1 as a regulator of chemokine function. ScientificWorldJournal 3: 1327–1331. 14755113 10.1100/tsw.2003.118 PMC5974740

[59] Carey DJ (1997) Syndecans: multifunctional cell-surface co-receptors. Biochem J 327 (Pt 1): 1–16. 10.1042/bj3270001 PMC1218755 9355727

[60] Dobra K, Nurminen M, Hjerpe A (2003) Growth factors regulate the expression profile of their syndecan co-receptors and the differentiation of mesothelioma cells. Anticancer Res 23: 2435–2444. 12894525

[61] Derksen PW, de Gorter DJ, Meijer HP, Bende RJ, van Dijk M, et al (2003) The hepatocyte growth factor/Met pathway controls proliferation and apoptosis in multiple myeloma. Leukemia 17: 764–774. 12682635 10.1038/sj.leu.2402875

[62] Kars MD, Iseri OD, Gunduz U (2010) Drug resistant breast cancer cells overexpress ETS1 gene. Biomed Pharmacother 64: 458–462. 20392592 10.1016/j.biopha.2010.01.008

[63] Takai N, Miyazaki T, Fujisawa K, Nasu K, Miyakawa I (2000) Expression of c-Ets1 is associated with malignant potential in endometrial carcinoma. Cancer 89: 2059–2067. 11066046 10.1002/1097-0142(20001115)89:10<2059::aid-cncr5>3.0.co;2-3

[64] Takai N, Miyazaki T, Nishida M, Nasu K, Miyakawa I (2002) c-Ets1 is a promising marker in epithelial ovarian cancer. Int J Mol Med 9: 287–292. 11836635 10.3892/ijmm.9.3.287

[65] Pap Z, Pavai Z, Denes L, Kovalszky I, Jung J (2009) An immunohistochemical study of colon adenomas and carcinomas: E-cadherin, Syndecan-1, Ets-1. Pathol Oncol Res 15: 579–587. 19253033 10.1007/s12253-009-9157-x

[66] Kopp JL, Wilder PJ, Desler M, Kim JH, Hou J, et al (2004) Unique and selective effects of five Ets family members, Elf3, Ets1, Ets2, PEA3, and PU.1, on the promoter of the type II transforming growth factor-beta receptor gene. J Biol Chem 279: 19407–19420. 14976186 10.1074/jbc.M314115200

[67] Russell L, Garrett-Sinha LA (2010) Transcription factor Ets-1 in cytokine and chemokine gene regulation. Cytokine 51: 217–226. 20378371 10.1016/j.cyto.2010.03.006

[68] Kumar R, Parsad D, Kanwar AJ, Kaul D (2011) Altered levels of Ets-1 transcription factor and matrix metalloproteinases in melanocytes from patients with vitiligo. Br J Dermatol 165: 285–291. 21428970 10.1111/j.1365-2133.2011.10324.x

[69] Ghosh S, Basu M, Roy SS (2012) ETS-1 Protein Regulates Vascular Endothelial Growth Factor-induced Matrix Metalloproteinase-9 and Matrix Metalloproteinase-13 Expression in Human Ovarian Carcinoma Cell Line SKOV-3. J Biol Chem 287: 15001–15015. 22270366 10.1074/jbc.M111.284034 PMC3340257

[70] Singh AK, Swarnalatha M, Kumar V (2011) c-ETS1 facilitates G1/S-phase transition by up-regulating cyclin E and CDK2 genes and cooperates with hepatitis B virus X protein for their deregulation. J Biol Chem 286: 21961–21970. 21515670 10.1074/jbc.M111.238238 PMC3121341

[71] Meng FK, Sun HY, Tan XY, Li CR, Zhou JF, et al (2011) Negative regulation of cyclin D3 expression by transcription factor c-Ets1 in umbilical cord hematopoietic cells. Acta Pharmacol Sin 32: 1159–1164. 21841808 10.1038/aps.2011.41 PMC4003296

[72] Schellings MW, Vanhoutte D, van Almen GC, Swinnen M, Leenders JJ, et al (2010) Syndecan-1 amplifies angiotensin II-induced cardiac fibrosis. Hypertension 55: 249–256. 20048198 10.1161/HYPERTENSIONAHA.109.137885

[73] Tian M, Schiemann WP (2009) The TGF-beta paradox in human cancer: an update. Future Oncol 5: 259–271. 19284383 10.2217/14796694.5.2.259 PMC2710615

[74] Marzo AL, Fitzpatrick DR, Robinson BW, Scott B (1997) Antisense oligonucleotides specific for transforming growth factor beta2 inhibit the growth of malignant mesothelioma both in vitro and in vivo. Cancer Res 57: 3200–3207. 9242450

[75] Suzuki E, Kim S, Cheung HK, Corbley MJ, Zhang X, et al (2007) A novel small-molecule inhibitor of transforming growth factor beta type I receptor kinase (SM16) inhibits murine mesothelioma tumor growth in vivo and prevents tumor recurrence after surgical resection. Cancer Res 67: 2351–2359. 17332368 10.1158/0008-5472.CAN-06-2389

[76] Stepp MA, Liu Y, Pal-Ghosh S, Jurjus RA, Tadvalkar G, et al (2007) Reduced migration, altered matrix and enhanced TGFbeta1 signaling are signatures of mouse keratinocytes lacking Sdc1. J Cell Sci 120: 2851–2863. 17666434 10.1242/jcs.03480

[77] Yue X, Li X, Nguyen HT, Chin DR, Sullivan DE, et al (2008) Transforming growth factor-beta1 induces heparan sulfate 6-O-endosulfatase 1 expression in vitro and in vivo. J Biol Chem 283: 20397–20407. 18503048 10.1074/jbc.M802850200 PMC2459296

[78] Yang JD, Sun Z, Hu C, Lai J, Dove R, et al (2011) Sulfatase 1 and sulfatase 2 in hepatocellular carcinoma: associated signaling pathways, tumor phenotypes, and survival. Genes Chromosomes Cancer 50: 122–135. 21104785 10.1002/gcc.20838 PMC3253341

[79] Kato M, Wang H, Bernfield M, Gallagher JT, Turnbull JE (1994) Cell surface syndecan-1 on distinct cell types differs in fine structure and ligand binding of its heparan sulfate chains. J Biol Chem 269: 18881–18890. 8034644

[80] Wang S, Ai X, Freeman SD, Pownall ME, Lu Q, et al (2004) QSulf1, a heparan sulfate 6-O-endosulfatase, inhibits fibroblast growth factor signaling in mesoderm induction and angiogenesis. Proc Natl Acad Sci U S A 101: 4833–4838. 15051888 10.1073/pnas.0401028101 PMC387334

[81] Guimond S, Maccarana M, Olwin BB, Lindahl U, Rapraeger AC (1993) Activating and inhibitory heparin sequences for FGF-2 (basic FGF). Distinct requirements for FGF-1, FGF-2, and FGF-4. J Biol Chem 268: 23906–23914. 7693696

[82] Sugaya N, Habuchi H, Nagai N, Ashikari-Hada S, Kimata K (2008) 6-O-sulfation of heparan sulfate differentially regulates various fibroblast growth factor-dependent signalings in culture. J Biol Chem 283: 10366–10376. 18281280 10.1074/jbc.M705948200

[83] Lai J, Chien J, Staub J, Avula R, Greene EL, et al (2003) Loss of HSulf-1 up-regulates heparin-binding growth factor signaling in cancer. J Biol Chem 278: 23107–23117. 12686563 10.1074/jbc.M302203200

[84] Lai JP, Chien J, Strome SE, Staub J, Montoya DP, et al (2004) HSulf-1 modulates HGF-mediated tumor cell invasion and signaling in head and neck squamous carcinoma. Oncogene 23: 1439–1447. 14973553 10.1038/sj.onc.1207258

[85] Lai JP, Chien JR, Moser DR, Staub JK, Aderca I, et al (2004) hSulf1 Sulfatase promotes apoptosis of hepatocellular cancer cells by decreasing heparin-binding growth factor signaling. Gastroenterology 126: 231–248. 14699503 10.1053/j.gastro.2003.09.043

[86] Dhoot GK, Gustafsson MK, Ai X, Sun W, Standiford DM, et al (2001) Regulation of Wnt signaling and embryo patterning by an extracellular sulfatase. Science 293: 1663–1666. 11533491 10.1126/science.293.5535.1663

[87] Ai X, Do AT, Lozynska O, Kusche-Gullberg M, Lindahl U, et al (2003) QSulf1 remodels the 6-O sulfation states of cell surface heparan sulfate proteoglycans to promote Wnt signaling. J Cell Biol 162: 341–351. 12860968 10.1083/jcb.200212083 PMC2172803

[88] Viviano BL, Paine-Saunders S, Gasiunas N, Gallagher J, Saunders S (2004) Domain-specific modification of heparan sulfate by Qsulf1 modulates the binding of the bone morphogenetic protein antagonist Noggin. J Biol Chem 279: 5604–5611. 14645250 10.1074/jbc.M310691200

[89] Bret C, Moreaux J, Schved JF, Hose D, Klein B (2011) SULFs in human neoplasia: implication as progression and prognosis factors. J Transl Med 9: 72. 21599997 10.1186/1479-5876-9-72 PMC3224561

[90] Dai Y, Yang Y, MacLeod V, Yue X, Rapraeger AC, et al (2005) HSulf-1 and HSulf-2 are potent inhibitors of myeloma tumor growth in vivo. J Biol Chem 280: 40066–40073. 16192265 10.1074/jbc.M508136200

[91] Rosen SD, Lemjabbar-Alaoui H (2010) Sulf-2: an extracellular modulator of cell signaling and a cancer target candidate. Expert Opin Ther Targets 14: 935–949. 20629619 10.1517/14728222.2010.504718 PMC3126665

[92] Bhattacharjee A, Richards WG, Staunton J, Li C, Monti S, et al (2001) Classification of human lung carcinomas by mRNA expression profiling reveals distinct adenocarcinoma subclasses. Proc Natl Acad Sci U S A 98: 13790–13795. 11707567 10.1073/pnas.191502998 PMC61120

[93] Nawroth R, van Zante A, Cervantes S, McManus M, Hebrok M, et al (2007) Extracellular sulfatases, elements of the Wnt signaling pathway, positively regulate growth and tumorigenicity of human pancreatic cancer cells. PLoS One 2: e392. 17460759 10.1371/journal.pone.0000392 PMC1849966

[94] He B, Lee AY, Dadfarmay S, You L, Xu Z, et al (2005) Secreted frizzled-related protein 4 is silenced by hypermethylation and induces apoptosis in beta-catenin-deficient human mesothelioma cells. Cancer Res 65: 743–748. 15705870

[95] Lee AY, Raz DJ, He B, Jablons DM (2007) Update on the molecular biology of malignant mesothelioma. Cancer 109: 1454–1461. 17348013 10.1002/cncr.22552

[96] Fox S, Dharmarajan A (2006) WNT signaling in malignant mesothelioma. Front Biosci 11: 2106–2112. 16720297 10.2741/1953

[97] Gonzalez AD, Kaya M, Shi W, Song H, Testa JR, et al (1998) OCI-5/GPC3, a glypican encoded by a gene that is mutated in the Simpson-Golabi-Behmel overgrowth syndrome, induces apoptosis in a cell line-specific manner. J Cell Biol 141: 1407–1414. 9628896 10.1083/jcb.141.6.1407 PMC2132788

[98] Theocharis AD, Seidel C, Borset M, Dobra K, Baykov V, et al (2006) Serglycin constitutively secreted by myeloma plasma cells is a potent inhibitor of bone mineralization in vitro. J Biol Chem 281: 35116–35128. 16870619 10.1074/jbc.M601061200

[99] Li XJ, Ong CK, Cao Y, Xiang YQ, Shao JY, et al (2011) Serglycin is a theranostic target in nasopharyngeal carcinoma that promotes metastasis. Cancer Res 71: 3162–3172. 21289131 10.1158/0008-5472.CAN-10-3557

[100] Henningsson F, Hergeth S, Cortelius R, Abrink M, Pejler G (2006) A role for serglycin proteoglycan in granular retention and processing of mast cell secretory granule components. FEBS J 273: 4901–4912. 17010166 10.1111/j.1742-4658.2006.05489.x

[101] Fitzgerald ML, Wang Z, Park PW, Murphy G, Bernfield M (2000) Shedding of syndecan-1 and -4 ectodomains is regulated by multiple signaling pathways and mediated by a TIMP-3-sensitive metalloproteinase. J Cell Biol 148: 811–824. 10684261 10.1083/jcb.148.4.811 PMC2169376

[102] Yu WH, Yu S, Meng Q, Brew K, Woessner JF Jr (2000) TIMP-3 binds to sulfated glycosaminoglycans of the extracellular matrix. J Biol Chem 275: 31226–31232. 10900194 10.1074/jbc.M000907200

[103] Leco KJ, Khokha R, Pavloff N, Hawkes SP, Edwards DR (1994) Tissue inhibitor of metalloproteinases-3 (TIMP-3) is an extracellular matrix-associated protein with a distinctive pattern of expression in mouse cells and tissues. J Biol Chem 269: 9352–9360. 8132674

[104] Nagase H, Visse R, Murphy G (2006) Structure and function of matrix metalloproteinases and TIMPs. Cardiovasc Res 69: 562–573. 16405877 10.1016/j.cardiores.2005.12.002

[105] Ramani VC, Pruett PS, Thompson CA, DeLucas LD, Sanderson RD (2012) Heparan sulfate chains of syndecan-1 regulate ectodomain shedding. J Biol Chem 287: 9952–9961. 22298773 10.1074/jbc.M111.330803 PMC3322978

[106] Ibrahim SA, Yip GW, Stock C, Pan JW, Neubauer C, et al (2012) Targeting of syndecan-1 by microRNA miR-10b promotes breast cancer cell motility and invasiveness via a Rho-GTPase- and E-cadherin-dependent mechanism. Int J Cancer 131: E884–896. 22573479 10.1002/ijc.27629

[107] Zimmermann P, David G (1999) The syndecans, tuners of transmembrane signaling. FASEB J 13 Suppl: S91–S100 10.1096/fasebj.13.9001.s91 10352150

[108] Hudson TJ, Anderson W, Artez A, Barker AD, Bell C, et al (2010) International network of cancer genome projects. Nature 464: 993–998. 20393554 10.1038/nature08987 PMC2902243

[109] Shannon P, Markiel A, Ozier O, Baliga NS, Wang JT, et al (2003) Cytoscape: a software environment for integrated models of biomolecular interaction networks. Genome Res 13: 2498–2504. 14597658 10.1101/gr.1239303 PMC403769

