# Supplementary figures and images for: Novel Genes and Pathways Modulated by Syndecan-1: Implications for the Proliferation and Cell-Cycle Regulation of Malignant Mesothelioma Cells
Source: PLoS One. 2012 Oct 29;7(10):e48091. doi: 10.1371/journal.pone.0048091 (PMC3483307; doi:10.1371/journal.pone.0048091)

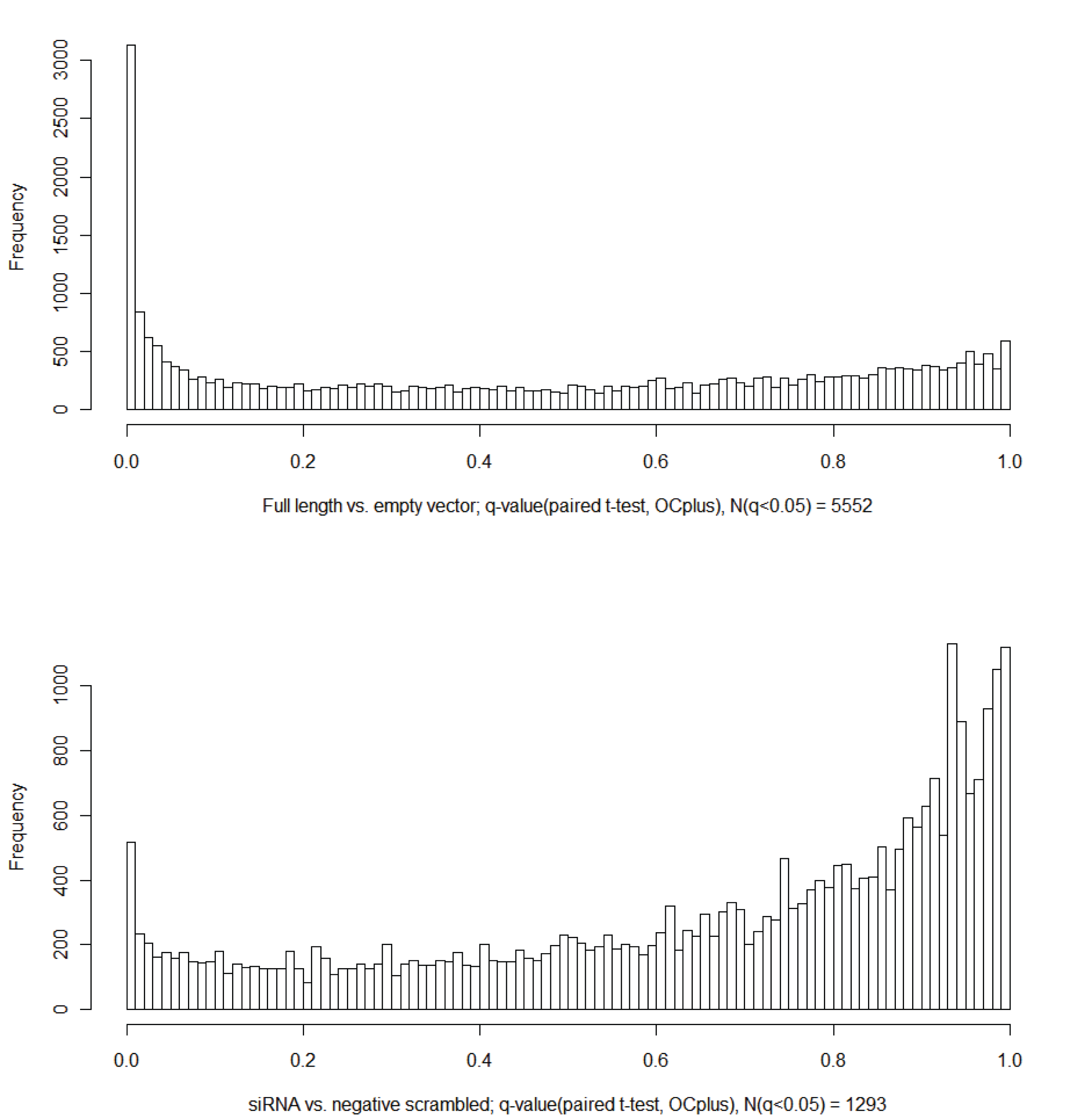

Supplement: Figure S1 — Distributions of false discovery rates (q) after differential expression analysis using paired t-test (R package OCplus). Legends at each plot denote comparison of sample and respective control and the total number of microarray probes that passed the criterion q≤0.05. Full-length represents genes from cells transfected with full-length syndecan-1 (i.e. -syndecan-1 overexpressed), empty vector represents genes from cells transfected with empty vector (control construct for overexpressed syndecan-1), siRNA corresponds to genes from cells with silenced syndecan-1, negative-scrambled corresponds to cells transfected with scrambled control construct for silenced cells. (TIF) [file pone.0048091.s001.tif]

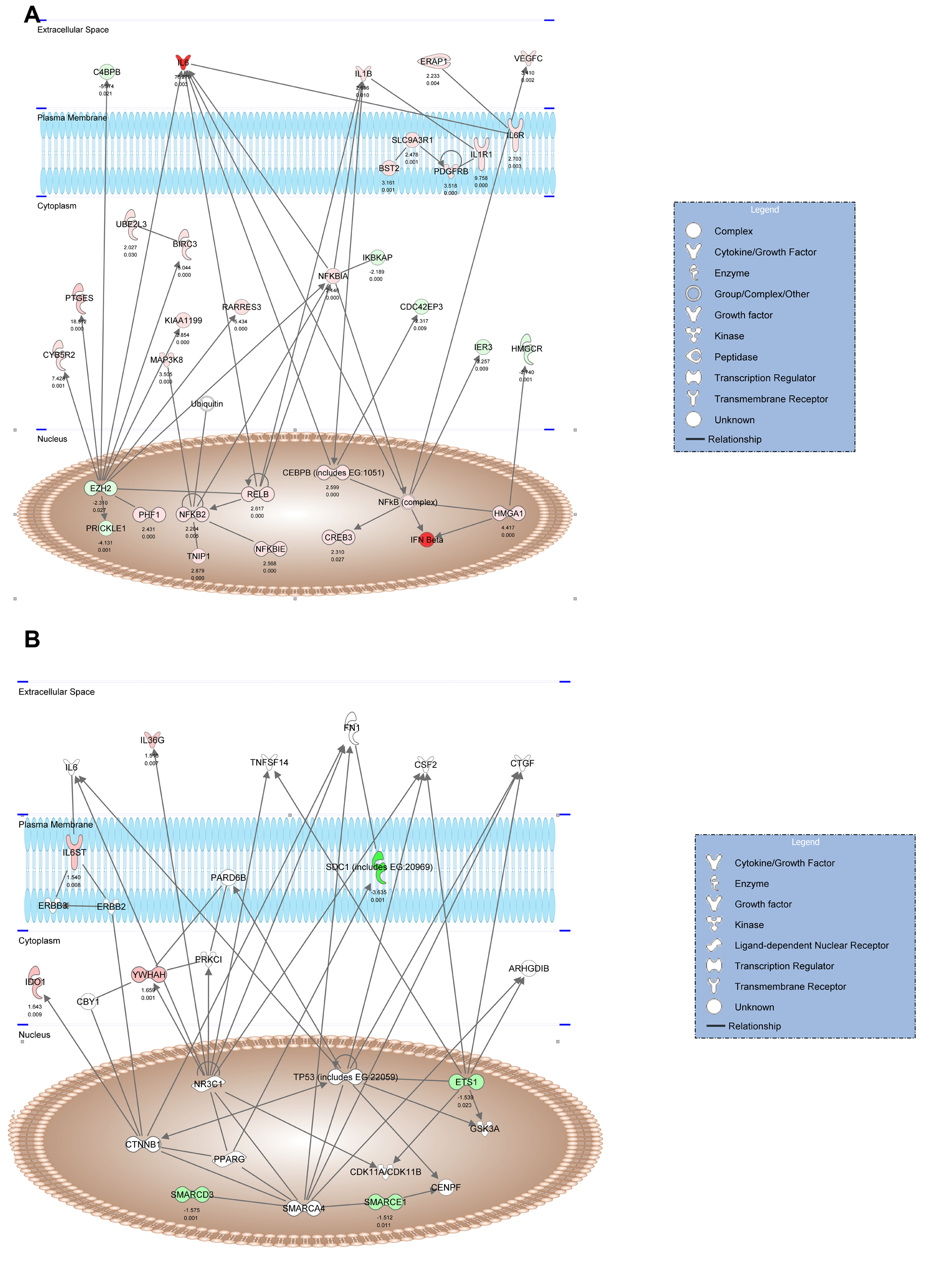

Supplement: Figure S2 — The most significant networks generated using IPA analysis. Networks were generated from genes altered by syndecan-1 overexpression (A) and silencing (B). The data set contains gene identifiers and corresponding expression values uploaded into the IPA application. A q cutoff of <0.05 was set to identify molecules whose expression was significantly differentially regulated. These molecules were overlaid onto a global molecular network in the Ingenuity Knowledge Base. Networks were then algorithmically generated based on their connectivity. Molecules are represented as nodes, and the biological relationship between two nodes is represented as an edge (line). All edges are supported by at least one reference from the literature, or from canonical information stored in the Ingenuity Knowledge Base. The intensity of the node color indicates the degree of up- (red) or downregulation (green). Nodes are displayed using various shapes that represent the functional class of the gene product. Edges are displayed with various labels that describe the nature of the relationship between the nodes. The associated functions correspond to Gene Expression, Inflammatory responses, Cancer for overexpression and Cellular development, Cell Growth and Proliferation, Gene Expression were associated to syndecan-1 silencing. For the graphical presentation the network analysis included the direct relationships only. (TIF) [file pone.0048091.s002.tif]
