# Supplementary material for: Novel Genes and Pathways Modulated by Syndecan-1: Implications for the Proliferation and Cell-Cycle Regulation of Malignant Mesothelioma Cells
Source: PLoS One. 2012 Oct 29;7(10):e48091. doi: 10.1371/journal.pone.0048091 (PMC3483307; doi:10.1371/journal.pone.0048091)
Supplement: Table S1 — siRNA construct sequences used. (DOCX) [file pone.0048091.s003.docx]

|  | siRNA construct sequences (sense, 5`-3`) |
| --- | --- |
| 1 | GACUUGUUUUUGCACAUGUUU |
| 2 | GCACCAUUCUGACUCGGUUUU |
| 3 | GCAGGUGCUUUGCAAGAUAUU |
