# Supplementary material for: Novel Genes and Pathways Modulated by Syndecan-1: Implications for the Proliferation and Cell-Cycle Regulation of Malignant Mesothelioma Cells
Source: PLoS One. 2012 Oct 29;7(10):e48091. doi: 10.1371/journal.pone.0048091 (PMC3483307; doi:10.1371/journal.pone.0048091)
Supplement: Table S2 — Primer sequences used for RT-PCR validation of differentially expressed genes and syndecan-1. (DOCX) [file pone.0048091.s004.docx]

| Gene | Primers (5´to 3´orientation) – sense/antisense |
| --- | --- |
| GAPDH | ACATCATCCCTGCCTCTACTGG/ AGTGGGTGTCGCTGTTGAAGTC [[20](#_ENREF_20)] |
| SDC1 | TCTGACAACTTCTCCGGCTC/CCACTTCTGGCAGGACTACA [[19](#_ENREF_19)] |
| ADAMTS5 | GCTACTGCACAGGGAAGAGG/ TGCATATTTGGGAACCCATT |
| EPYC | CCAGAATTGCCAACCACTTT/CGTGCATTTCCAGAATGTTG |
| ETS1 | TGGAGTCAACCCAGCCTATC/TCTGCAAGGTGTCTGTCTGG |
| FBLN5 | GACGGATATTGGCTTCTGGA/GGCAGATGAAAGAGCCGTAG |
| FN1 | CCAACCTACGGATGACTCGT/GCTCATCATCTGGCCATTTT |
| GPC6 | CAACATTGAGTCGGTCATGG/ATTGTAGGGCCTGAAACGTG |
| IL33 | CAAAGAAGTTTGCCCCATGT/AAGGCAAAGCACTCCACAGT |
| IL6 | AGGAGACTTGCCTGGTGAAA/CAGGGGTGGTTATTGCATCT |
| INHBA | TTTCTGTTGGCAAGTTGCTG/CGGGTCTCTTCTTCAAGTGC |
| LRRC7 | CGAGGCCTACTCCTGTGAAG/ACGGGGTTTTCTGAAGAGGT |
| LUM | CCTGAAAGCTACCCAAGTGC/AAAAGAGCCCAGCTTTGTGA |
| PDGFRA | GAAGCTGTCAACCTGCATGA/CTTCCTTAGCACGGATCAGC |
| RARRES | GAGCGCTACAACCCAGAGTC/GAAAGCCAAATCCCAGATGA |
| SLAMF7 | GCACCTGTGTGACCAATCTG/AAGGATGGGGCTTGAGAAGT |
| SRGN | CGCTGCAATCCAGACAGTAA/CCTGTTCCATTTCCGTTAGG |
| SULF1 | AAGGTTAATCAGCCCCGTCT/ACCAAGAACCCGTCACTTTG |
| SULT1B1 | GGTTATCCCATGACCTGTGC/CCAGGGAGAGTCATTTCCAA |
| SULT1E1 | AGGGTGATGTGGAAAAGTGC/GGCAGGAAGAAGTTCAGGTG |
| TGFΒ2 | TGCTTTGGCTTTCTGGTTCT/TTTGTTTGTGGTGCAGTGGT |
| TNXB | CTCTGGGTGTGTGGTCAATG / CCCTCCCCAAATAAGTGGAT |
