# Supplementary material for: Novel Genes and Pathways Modulated by Syndecan-1: Implications for the Proliferation and Cell-Cycle Regulation of Malignant Mesothelioma Cells
Source: PLoS One. 2012 Oct 29;7(10):e48091. doi: 10.1371/journal.pone.0048091 (PMC3483307; doi:10.1371/journal.pone.0048091)
Supplement: Table S3 — The rate of apoptotic cells in the syndecan-1 silenced cells compared to the scrambled siRNA control. Apoptosis was measured by flow cytometry using Annexin-V-FITC and Propidium iodide (PI) staining, 24 and 48 hours after syndecan-1 silencing. The results are mean of three independent experiments ±SD. No significant changes were recorded in the rate of apoptotic cells. (DOCX) [file pone.0048091.s005.docx]

| TIME | APOPTOSIS | SCRAMBLED CONTROL  (% of cells) | SILENCED SYNDECAN-1  (% of cells) |
| --- | --- | --- | --- |
| 24h | EARLY APOPTOSIS | 1.56±0.60 | 1.63±0.69 |
|  | LATE APOPTOSIS | 2.97±0.45 | 2.32±0.49 |
| 48h | EARLY APOPTOSIS | 1.27±0.24 | 1.74±0.49 |
|  | LATE APOPTOSIS | 5.48±1.99 | 5.25±2.11 |
