# Supplementary material for: Novel Genes and Pathways Modulated by Syndecan-1: Implications for the Proliferation and Cell-Cycle Regulation of Malignant Mesothelioma Cells
Source: PLoS One. 2012 Oct 29;7(10):e48091. doi: 10.1371/journal.pone.0048091 (PMC3483307; doi:10.1371/journal.pone.0048091)
Supplement: Table S4 — Selected functional categories affected by syndecan-1 overexpression. Genes are grouped by using GO terms. FC = fold changes, all differences correspond to q≤0.05. (DOCX) [file pone.0048091.s006.docx]

| GENE ID | GENE NAME | FC | |
| --- | --- | --- | --- |
| a) CHEMOKINES/ILs and IL receptors | | | |
| CCL19 | chemokine (C-C motif) ligand 19 | 1,55 | |
| CCL2 | chemokine (C-C motif) ligand 2 | 1,70 | |
| CXCL1 | chemokine (C-X-C motif) ligand 1 (melanoma growth stimulating activity) | 1,55 | |
| CXCL16 | chemokine (C-X-C motif) ligand 16 | 2,70 | |
| IL1R1 | interleukin 1 receptor, type I | 9,76 | |
| IL1A | interleukin 1, alpha | 3,36 | |
| IL1B | interleukin 1, beta | 2,64 | |
| IL4R | interleukin 4 receptor | 2,53 | |
| IL6 | interleukin 6 (interferon, beta 2) | 76,28 | |
| IL6R | interleukin 6 receptor | 2,70 | |
| IL7R | interleukin 7 receptor | 5,19 | |
| IL8 | interleukin 8 | 10,04 | |
| IL12A | interleukin 12A (natural killer cell stimulatory factor 1, p35) | 1,82 | |
| IL20RB | interleukin 20 receptor beta | 2,13 | |
| IL32 | interleukin 32 | 1,67 | |
| IL33 | interleukin 33 | 277,7 | |
| b) GROWTH FACTORS AND GROWTH FACTOR RECEPTORS | | |  |
| EGF | epidermal growth factor (beta-urogastrone) | 1,54 | |
| EGFR | epidermal growth factor receptor (erythroblastic leukemia viral (v-erb-b) | 2,50 | |
| FGF18 | fibroblast growth factor 18 | -1,82 | |
| FGFR2 | fibroblast growth factor receptor 2 (keratinocyte growth factor receptor) | 6,33 | |
| PDGFC | platelet derived growth factor C | -2,88 | |
| PDGFRA | platelet-derived growth factor receptor, alpha polypeptide | 11,1 | |
| PDGFRB | platelet-derived growth factor receptor, beta polypeptide | 3,52 | |
| INHBA | inhibin, beta A | -59,4 | |
| ACVR1B | activin A receptor, type IB | 2,70 | |
| TGFB2 | transforming growth factor, beta 2 | -13,1 | |
| TGFB3 | transforming growth factor, beta 3 | -2,99 | |
| TGFBR1 | transforming growth factor, beta receptor I (activin A receptor type II-like kinase,) | -4,46 | |
| TGFBR2 | transforming growth factor, beta receptor II (70/80kDa) | 1,91 | |
| BMP1 | bone morphogenetic protein 1 | -1,70 | |
| BMPR1A | bone morphogenetic protein receptor, type IA | -1,71 | |
| BMPR2 | bone morphogenetic protein receptor, type II (serine/threonine kinase) | -2,29 | |
| VEGFA | vascular endothelial growth factor A | 2,42 | |
| VEGFB | vascular endothelial growth factor B | 1,57 | |
| VEGFC | vascular endothelial growth factor C | 3,41 | |
| KDR | kinase insert domain receptor (a type III receptor tyrosine kinase, VEGFR2) | 1,97 | |
| NRP1 | neuropilin 1 | 4,54 | |
| NRP2 | neuropilin 2 | 18,00 | |
| MDK | midkine (neurite growth-promoting factor 2) | -1,69 | |
| BDNF | brain-derived neurotrophic factor | -3,13 | |
| NRG4 | neuregulin 4 | -2,18 | |
| NTF3 | neurotrophin 3 | 16,84 | |
| TYMP | thymidine phosphorylase (endothelial cell growth factor) | 1,72 | |
| LIF | leukemia inhibitory factor (cholinergic differentiation factor) | 2,34 | |
| HBEGF | heparin-binding EGF-like growth factor | -4,21 | |
| IGF1R | insulin-like growth factor 1 receptor | -2,96 | |
| CTGF | connective tissue growth factor | -3,38 | |
| PTK7 | PTK7 protein tyrosine kinase 7 | -1,73 | |
| c) PROTEOGLYCANS | | | |
| BGN | biglycan | -6.21 | |
| LUM | lumican | -16.51 | |
| DCN | decorin | -6.83 | |
| EPYC | epiphycan | -8.97 | |
| SDC2 | SDC2 | -3 | |
| GPC3 | glypican 3 | 1.82 | |
| GPC6 | glypican 6 | -9.26 | |
| SRGN | serglycin | 52.87 | |
| PRG4 | proteoglycan 4 | 7.42 | |
| PRG2 | proteoglycan 2, bone marrow (natural killer cell activator) | 2.03 | |
| CSPG4 | chondroitin sulfate proteoglycan 4 | 3.2 | |
| HSPG2 | heparan sulfate proteoglycan 2 | -1.56 | |
| d) PROTEASES/PROTEASE INHIBITORS | | | |
| MMP15 | matrix metallopeptidase 15 (membrane-inserted) | 2.54 | |
| MMP16 | matrix metallopeptidase 16 (membrane-inserted) | -7.97 | |
| MMP24 | matrix metallopeptidase 24 (membrane-inserted) | -6.6 | |
| ADAM2 | ADAM metallopeptidase domain 2 (fertilin beta) | 2.35 | |
| ADAM18 | ADAM metallopeptidase domain 18 | 2.47 | |
| ADAM23 | ADAM metallopeptidase domain 23 | -6.57 | |
| ADAMTS5 | ADAM metallopeptidase with thrombospondin type 1 motif, 5 (aggrecanase-2) | -26.78 | |
| TIMP3 | TIMP metallopeptidase inhibitor 3 | -3.35 | |
| e) SULFATASES/SULFOTRANSFERASES | | | |
| SULF1 | sulfatase 1 | -52.32 | |
| HS2ST1 | heparan sulfate 2-O-sulfotransferase 1 | 1.55 | |
| HS6ST1 | heparan sulfate 6-O-sulfotransferase 1 | -3.53 | |
| SULT1E1 | sulfotransferase family 1E, estrogen-preferring, member 1 | -307.61 | |
| SULT1A1 | sulfotransferase family, cytosolic, 1A, phenol-preferring, member 1 | 2.77 | |
| SULT1A2 | sulfotransferase family, cytosolic, 1A, phenol-preferring, member 2 | 4.21 | |
| SULT1A3 | sulfotransferase family, cytosolic, 1A, phenol-preferring, member 3 | 1.86 | |
| SULT1B1 | sulfotransferase family, cytosolic, 1B, member 1 | -179.35 | |
| ARSA | arylsulfatase A | 2.53 | |
| ARSJ | arylsulfatase family, member J | 2.08 | |
| SGSH | N-sulfoglucosamine sulfohydrolase (sulfamidase) | 1.72 | |
